# “Live” cell shipment—a forward-looking transport option for cryo-sensitive cell-based therapies

**DOI:** 10.3389/fbioe.2025.1706927

**Published:** 2025-12-09

**Authors:** Lutz Roßbach, Thea Eichenberg, Nicole Pietzsch, Paul Franz, Stephan Fricke, Robin Sieg, Anna Dünkel, Kathrin Sabine Adlkofer

**Affiliations:** 1 Fraunhofer Institute for Cell Therapy and Immunology, Leipzig, Germany; 2 Cellbox Solutions GmbH, Hamburg, Germany

**Keywords:** live cell shipment, NK cells, cell-based therapies, regenerative medicine, cancer

## Abstract

Cell therapies, which use the transplantation or manipulation of cells to treat diseases, are highly promising for conditions such as cancer, autoimmune diseases, and genetic disorders. Their success depends on the quality and viability of the administered cell products, which is especially critical for patients already weakened by chemotherapy or radiation. In such cases, ensuring high-quality cells that can engraft, proliferate, and function properly is essential for improving outcomes and survival. Natural killer (NK) cells are strong candidates for cancer treatment due to their innate cytotoxic activity without the side effects of graft-versus-host disease (GvHD). However, cryopreservation has significantly hindered their clinical application, as it reduces both survival and function. Current “off-the-shelf” allogeneic NK therapies, therefore, face limitations because freezing and thawing lead to cell loss and impaired activity. To address this challenge, we evaluated live shipment of NK cells using the Cellbox™ Shipper Flight, a transportable incubator maintaining 37°C and 5% CO_2_, versus cryopreservation and static laboratory incubation. Three independent NK cell batches, expanded under clinically compliant conditions, were tested. Optimized cryopreservation yielded 78% ± 9% of initial cell counts with >96% viability immediately after thawing. However, phenotypic analyses revealed a loss of NK markers, reduced cytotoxic activity, and an additional 50% ± 1% loss during 3 days of recovery, indicating that nearly half of the thawed cells were non-functional. In contrast, NK cells shipped live in the Cellbox™ retained an unaffected phenotype and cytotoxic function. After transport, cell counts recovered, and proliferation was observed during 3 days of static recultivation. Overall, live shipment delivered 2.5 times more functional NK cells after recovery than cryopreservation. These findings demonstrate that shipping NK cells in culture using Cellbox™ is both feasible and advantageous, preserving cell function and ensuring a full therapeutic dose. By contrast, cryopreservation not only reduces immediate viability but also compromises long-term recovery and functionality, potentially limiting clinical efficacy. Mobile incubators like Cellbox™, therefore, represent a critical advance, enabling the reliable transport of living therapeutic cells and unlocking the full potential of cell therapies for patient benefit.

## 1 Introduction

The research and development of novel cell-based approaches to treat a wide range of diseases or tissue and organ damage is a worldwide endeavor. Cell-based products, genetically engineered or not, have in common that they are extremely complex and demanding to manufacture and therefore are produced in specialized facilities (Hopewell et al., 2024; Myles and Church, 2022; Saultz and Otegbeye, 2023). In any case, the benefit–risk balance of cryopreservation must be systematically assessed, and the freezing and thawing processes carefully developed for each product (Meneghel et al., 2020; Thirumala et al., 2009). For some products, like complex 3D tissue-like structures made of several cell types or highly differentiated iPSC-derived cells, the standard slow freezing cryopreservation does not seem to be an option (Taylor et al., 2019; Bahsoun et al., 2019). Likewise, cryopreservation of natural killer (NK) cells has been challenging as it resulted in loss of viability or function and initial clinical trials using cryopreserved NK cells showed poor *in vivo* recovery, survival, and function (Miller et al., 2014; Lapteva et al., 2012; Damodharan et al., 2020; Mark et al., 2020) leading some investigators to ship “fresh,” that is, unfrozen, NK cells to clinical centers (Szmania et al., 2015) and some others to set up post-thaw IL-2-dependent recovery protocols (Berg et al., 2009). NK cells are seen as excellent candidates for adoptive cancer immunotherapy based on their inherent ability to recognize and kill virally infected, stressed, or cancerous cells without prior sensitization or antigen presentation. They have already shown early clinical evidence of efficacy in hematologic malignancies with low risk for cytokine release syndrome and graft-versus-host disease (GvHD) in allogeneic settings (Lamb et al., 2021). Efforts made to develop methods to use adoptive NK cells, including CAR-NK cells, in immunotherapeutic treatments for cancer might fall short in the absence of options for the shipment of the final product that can support, in conjunction with other approaches, long survival and persistence of NK cells *in vivo* (Berrien-Elliott et al., 2023; Laskowski et al., 2022; Myers and Miller, 2021).

With the aim of identifying shipping modalities more appropriate to NK cell physiology and function, we tested a 2-day shipment in a transportable incubator (the Cellbox™ Shipper Flight) at 37 °C and 5% CO_2_ compared to state-of-the-art cryopreservation and thawing procedures and static incubation in a laboratory incubator under optimal conditions. Unlike most current active configuration shippers aimed at the cold chain, shippable passive CO_2_ incubators have recently been developed to transport living cell and tissue cultures under laboratory conditions in a so-called warm chain. This includes transports of living cells at refrigerated temperature (2 °C–8 °C), via room temperature (15 °C–25 °C) up to the typical cultivation temperature (e.g., 37 °C). The Cellbox™ device is the only active system with its own integrated CO_2_ source and an international flight allowance.

The benefit of a “live” shipment over cryopreservation was demonstrated using a recurring quality control assessing cell numbers, viability, culture metabolites, cell phenotype, and cytotoxic function with a clear statement based on the analysis of cells post-thawing and after a recovery period of 3 days. These analyses clearly showed that live shipment preserved NK cell viability and functionality better than cryopreserved NK cells.

## 2 Materials and Methods

### 2.1 Ethical statement

Buffy coats from healthy donors were obtained after informed consent from the Institute for Transfusion Medicine of the Leipzig Medical Center. The study was approved by the local Ethics Committee (327/22-ek) of the University of Leipzig.

### 2.2 NK cell isolation

Donor buffy coats were received approximately 16 h after blood donation from the University of Leipzig and kept at room temperature until further processing. Peripheral blood mononuclear cell (PBMC) isolations were performed via density centrifugation with Histopaque-1077 (Sigma-Aldrich, Cat. No.: 10771-500 ML) and SepMate 50 tubes (STEMCELL Technologies, Cat. No.: 85450) centrifuged at 1200 g for 20 min. NK cell isolation was then performed according to the user manual using a STEMCELL Technologies NK cell isolation kit (Cat. No.: 17955) and an EasySep 50 Magnet (STEMCELL Technologies, Cat. No.: 18002).

### 2.3 Primary NK cell culture

NK cells were seeded at 3 × 10^7^ cells/well in 8 mL of media in a G-Rex® 24-well plate from WilsonWolf (Cat. No.: 80192M) (2 cm^2^) and 1–2 × 10^8^ cells/well in 35 mL of medium in a G-Rex® 6-well plate (10 cm^2^). Media changes were performed every 2 or 3 days. Seventy-five percent of the media was changed each time, and the cells were counted afterward. NK MACS® media from Miltenyi Biotec (Cat. No.: 130-114-429) as basal media was used with 1% supplement (according to the Miltenyi Biotec user manual), 5% human AB serum from Bio&SELL (Cat. No.: HUAB.SE.010), and IL-2 (500 U/mL; Cat. No.: 130-097-142) and IL-15 (140 U/mL; Cat No.: 130-095-764) from Miltenyi Biotec. The NK cells were expanded and activated without feeder cells. The incubation was performed at 37 °C and 5% CO_2_.

For the bag test, the isolated NK cells were expanded up to 4 × 10^8^ NK cells per donor in a G-Rex® System from WilsonWolf for up to 21 days. For a G-Rex® 24-well plate, a maximum of 3 × 10^7^ viable NK cells were seeded in 8 mL per well. When viable cell counts reached 1 × 10^8^ per G-Rex® 24-well plate (usually after four or five passages), NK cells were pooled and seeded into a G-Rex® 6-well plate using 35 mL supplemented NK MACS® media until harvest. NK cells were then split into three different conditions and seeded at 1.4 × 10^8^ viable NK cells in 35 mL of supplemented NK MACS media. OriGen Biomedical PermaLife™ Cell Culture Bag (Cat. No.: PL70-2G) (Bag A) and Miltenyi Biotec MACS® GMP Cell Differentiation Bag - 100 (Cat. No.: 170-076-400) (Bag B) were used for a three-day cultivation test and compared to a 6-well G-Rex® plate used as a control. NK cells in the cultivation test were analyzed regarding their proliferation capacity, viability, surface marker expression using flow cytometry, and cytotoxic potential.

For the transport testing, the isolated NK cells were expanded at least to 6.4 × 10^8^ NK cells per donor in a G-Rex® System up to 21 days using the culture conditions described above. The final NK cell culture was split into four separate batches, each 1.47% × 10^8^% ± 15% viable NK cells in 35 mL supplemented NK MACS® media (4.2 × 10^6^ NK cells/mL per condition). One-fourth was cryopreserved (see below). Two control conditions were incubated in the static laboratory incubator: one well of a G-Rex® 6-well plate and one PermaLife™ Cell Culture Bag from OriGen (Cat. No.: PL70-2G, Bag A). The last fourth of the culture was used to fill a second PermaLife™ Cell Culture Bag from OriGen (also Bag A) for further packaging into a Cellbox™ Shipper Flight.

Immediately after shipment or thawing, NK cells from all four conditions were assayed for cell counts, viability, phenotype, exhaustion markers, and cytotoxic activity. Sterility, *Mycoplasma*, and endotoxins were assayed for the Cellbox™ condition only. Metabolites and pH were measured in all culture supernatants (not for cryopreserved cells).

NK cells were then seeded into a G-Rex® 24 plate at a concentration of 3 × 10^7^ viable NK cells per well with 8 mL of supplemented NK MACS® media and incubated for 72 h at 37 °C and 5% CO_2_ in a static incubator for an additional 3-day period.

### 2.4 Cryopreservation

For cryopreservation, 1.4 × 10^8^ NK cells were centrifuged and resuspended in 14 mL cold CryoStor® CS10 (Biolife Solutions; Cat. No.: 210202) and added to 1.8 mL cryo vials (1 × 10^7^ NK cells per mL and per vial). Vials were cryopreserved in a controlled rate freezer (CRF, Thermo Scientific, CryoMed™ Controlled Rate Freezer, Type: 7455) according to the program outlined below (Table 1). One vial, filled with 1 mL of CS10, was used as a reference vial with a probe for temperature recording during the freezing process. The monitoring of the cryopreservation was performed via online data monitoring.

**TABLE 1:** Controlled rate freezer program used to cryopreserve NK cell vials.

Program step	Target temperature	Ramp	Reference
1	+4 °C	NA	Chamber
2	−4 °C	1 °C/min	Sample
3	−40 °C	25 °C/min	Chamber
4	−12 °C	10 °C/min	Chamber
5	−40 °C	1 °C/min	Chamber
6	−90 °C	10 °C/min	Chamber (end)

### 2.5 Shipment in the Cellbox™

In vitro expanded NK cells (1.47 × 10^8^ ± 0.22 × 10^8^ cells), suspended in 35 mL supplemented NK MACS® media, were placed into one OriGen PermaLife™ Cell Culture Bag (Bag A) and used for the transport in the Cellbox™ Shipper Flight. Together with a pad of liquid-absorbing material, the cell bag was placed into a breathable, watertight Tyvek envelope as a secondary container. The wrapped cell bag was stored in the laboratory incubator (37 °C, 5% CO_2_) for further use. When the empty Cellbox™ incubation chamber reached 37 °C, dry ice pellets (as a source of CO_2_) were placed into the appropriate reservoir in the Cellbox™ Shipper, and the setpoint of 5% CO_2_ was defined. Once the target CO_2_ concentration was reached, the wrapped cell bag was transferred into the incubation chamber of the Cellbox™. Reticulated foam cushions of 5 mm, 10 mm, 15 mm, and 20 mm thickness from Architekturbedarf.de (Cat. Nos: 542-365002, 542-365003, 542-365004, and 542–365005) were used to absorb shocks and stabilize the bag within the incubation chamber. Transport was performed by road and train back and forth from Leipzig to Norderstedt (approximately 850 km one way) with an overnight storage at Cellbox Solutions facilities in Norderstedt. After 44 h of transport, the Cellbox™ was opened, the wrapped cell bag was taken out of the incubation chamber, and its integrity was checked by visual inspection. The cell bag was stored in the laboratory incubator at 37 °C and 5% CO_2_ until further processing. Data were collected every minute by the Cellbox™ and subsequently stored internally. A smartphone-based interface via an app enables data export after transportation using Bluetooth. The following parameters were monitored: incubation temperature, ambient temperature and pressure, incubation CO_2_ concentration, remaining CO_2_ in the dry ice reservoir, battery level, tilts in the x- and y-axes, vibrations, and accelerations.

### 2.6 Viable cell counts and cell viability

All cell concentrations and viabilities were measured with the NC-202 according to the ChemoMetec user manual. In case the sample contained more than 1 × 10^7^ cells/mL (upper range), samples were diluted 10-fold with culture media (e.g., 20 μL original cell suspension in 180 µL NK MACS® media).

Afterward, the cumulative population doublings (CPD) and expansion folds were calculated (Spohn et al., 2021).

Equation to calculate the CPD:

$${CPD}_{t0}={CPD}_{t-1}+3.322\;*\left[{\mathrm{LOG}\left(viable\;cc\right)}_{t0}-{\mathrm{LOG}\left(viable\;cc\right)}_{t-1}\right].$$

Here,

CPD_t0_ = current CPD.

CPD_t−1_ = CPD of the previous time point.

LOG (viable cc)t0 = Log_10_ [viable cell count of current time point].

LOG (viable cc)t−1 = Log_10_ [viable cell count of previous time point].

### 2.7 pH measurement

All pH measurements were performed using cell suspension or supernatant and a SevenEasy pH meter by Mettler Toledo in accordance with the user manual.

### 2.8 Glucose and lactate concentration measurements

The measurement of glucose (GLC) and lactate (LAC) was performed by using cell culture supernatants and a Cedex Bio HT from Roche Diagnostics in accordance with the user manual.

### 2.9 Measurement of NK cell antigen receptors

Due to their natural function, NK cells express several different surface molecules/receptors that can be used to monitor the phenotype of the cells. The expression pattern of certain receptors changes during the development of NK cells and can provide information on the cells’ overall fitness and their level of activation or exhaustion. We looked at the surface expression of NKG2D because it serves as a major recognition receptor for the detection and elimination of transformed and infected cells by triggering NK cell cytotoxicity and cytokine secretion. The ligands for NKG2D are structural homologs of MHC class I molecules (Siemaszko et al., 2021) that are frequently expressed on pathophysiologically stimulated cells, while they are absent or expressed at a very low level in normal cells. Study of the expression of DNAM1 and three activating molecules that belong to natural cytotoxicity receptors (NCRs), namely, NCR1 (*NKp46*), NCR2 (*NKp44*), and NCR3 (*NKp30*), was also part of this study, as they all function in the recognition of tumor cells and induction of cytotoxic effects (Siemaszko et al., 2021).

For phenotypic analysis, PBMCs and NK cells were washed once with autoMACS® Running Buffer (Miltenyi Biotec, Cat. No.: 130-091-221) and incubated with five times diluted FcR Blocking Reagent (Miltenyi Biotec, Cat. No. = 130-059-901) for 10 min at 4 °C. For extracellular staining, cells were incubated at room temperature for 30 min in the dark with the monoclonal antibodies (BD Biosciences or BioLegend) for immune status, receptor, and exhaustion panel, respectively. To exclude dead cells from the analysis, cells were stained with BD Horizon™ Fixable Viability Stain 780 simultaneously with the antibodies. After washing once with autoMACS® Running Buffer, cells were analyzed with a 10-color Navios EX flow cytometer (Beckman Coulter). Acquired data were analyzed using Kaluza Analysis 2.1 software.

For the flow cytometry, the following pre-conjugated antibodies were used.

### 2.10 Immune status panel

CD3-FITC (BD Biosciences, Cat No.: 555332), CD8a-PE (BioLegend, Cat No.: 301008), CD14-PerCP (BD Biosciences, 345786), CD56-PE/Cy7 (BD Biosciences, Cat. No.: 335826), CD19-APC (BD Biosciences, Cat No.: 345791), CD4-APC/H7 (BD Biosciences, Cat. No.: 560158), CD16-BV421 (BD Biosciences, Cat. No.: 562874), and CD45-V500 (BD Biosciences, Cat. No.: 560777).

### 2.11 Receptor panel

CD56-PE (BioLegend, Cat. No.: 362508), NKp30-PerCP/Cy5.5 (BioLegend, Cat. No.: 325216), NKp44-PE/Cy7 (BioLegend, Cat. No.: 325116), NKp46-APC (BioLegend, Cat. No.: 331918), NKG2D-BV421 (BioLegend, Cat. No.: 320822), DNAM1-BV510 (BioLegend, Cat. No.: 338330), CD16-AF700 (BioLegend, Cat. No.: 302026), and BD Horizon™ Fixable Viability Stain 780 (BD Biosciences, Cat. No.: 565388).

### 2.12 Exhaustion panel

CD56-AF700 (BioLegend, Cat. No.: 392417), CD3-BV421 (BioLegend, Cat. No.: 300434), PD-1-APC (BioLegend, Cat. No.: 621610), TIM3-PerCp/Cy5.5 (BioLegend, Cat. No.: 345016), LAG-3-FITC (BioLegend, Cat. No.: 369308), CD16-PE/Cy7 (BioLegend, Cat. No.: 302016), and BD Horizon™ Fixable Viability Stain 780 (BD Biosciences, Cat. No.: 565388).

For each staining, 5 × 10^5^ cells were washed once with autoMACS® Running Buffer. FcR-block reagent (1:5) was also diluted with autoMACS® Running Buffer. Antibody cocktails were diluted in the prepared FcR-Block reagent. The negative controls consisted of 5 × 10^5^ unstained cells resuspended in FcR-Block reagent. In the receptor panel, fluorescence minus one (FMO) controls were prepared for the markers NKp30, DNAM-1, and NKG2D based on the overlapping of the spectral ranges of the fluorochromes PerCP/Cy5.5, BV510, and BV421. For each of these controls, one of the above-mentioned markers with the corresponding fluorochrome was omitted from one control, and the measurement signal of the other fluorochrome was determined. The measurement signals of all possible combinations of these fluorochromes were then analyzed by overlapping. The combined measurement signal was used to analyze NKp30, DNAM-1, and NKG2D.

FMOs for the Exhaustion Panel were prepared using the LAG3, PD-1, and TIM-3 analog described above. After an incubation of 30 min at RT, the samples were washed twice and resuspended in 400 µL autoMACS® Running Buffer for flow cytometry measurement.

### 2.13 Measurement of NK cell activity by calcein release assay

NK cell killing activity was determined by a calcein-acetoxymethyl (calcein AM) release assay. In all cocultivation assays, RPMI1640 media (Thermo Fisher Scientific, Cat. No: 21875034) was used and supplemented with 10% FBS (Thermo Fisher Scientific, Cat. No.: A4766801). K-562 used as target cells were loaded with Calcein AM (Thermo Fisher Scientific, Cat. No.: C3100MP), and NK cell-mediated cytotoxicity was assessed after 4 h of cocultivation. Spontaneous calcein release was assessed using labeled K-562 w/o NK cell cocultivation. Therefore, 1 × 10^4^ calcein-labeled K-562 cells in 50 µL were mixed with 150 µL RPMI1640 media (10% FBS) only. For the maximum release (100% of target cell death), 1 × 10^4^ calcein-labeled K-562 in 50 µL per well were mixed with 150 µL of a 2% Triton X-100 solution (Sigma-Aldrich, Cat. No: 28817295) in RPMI1640 (10% FBS) media. The assay was performed in technical triplicate, and the emission (535 nm) of each sample was measured by an Infinite 200 PRO plate reader (Tecan). The relative calcein release was determined by the following equation for each sample (Yamashita et al., 2016):

$$\text{relative calcein release }\left[\%\right]=\left(\text{sample release}-\text{spontaneous release}\right)/\left(\text{maximal release}-\text{spontaneous release}\right)\times 100\%$$

Four different NK cell (effector) versus K-562 cell (target) ratios, that is, E:T (Effector:Target) ratios, of 5, 1, 0.5, and 0.1 were tested. Calcein from labeled K-562 cells is released into the co-culture supernatant as cells are killed by NK cells. Calcein was then quantified in the supernatant.

### 2.14 Microscopic observation

Morphology of single cells or NK cell clusters was assessed by using a Zeiss microscope with standard magnifications ×100 and ×400. NK cell cluster sizes were determined using Zeiss ZEN Software.

### 2.15 Sterility, endotoxin, and *Mycoplasma* testing

Sterility testing was performed on 1 mL of cell suspension supernatant in 40 mL iAST (bioMérieux, Cat. No.: 259786) (aerobic) and in 40 mL iNST (bioMérieux, Cat. No.: 259785) (anaerobic) test media. Test vials were incubated for 2 weeks in the BacTAlert (bioMérieux). Endotoxin testing was performed by the colorimetric LAL-Test according to the European Pharmacopeia. *Mycoplasma* detection was performed by RT-PCR.

## 3 Results

### 3.1 The Cellbox™ system

**FIGURE 1:**
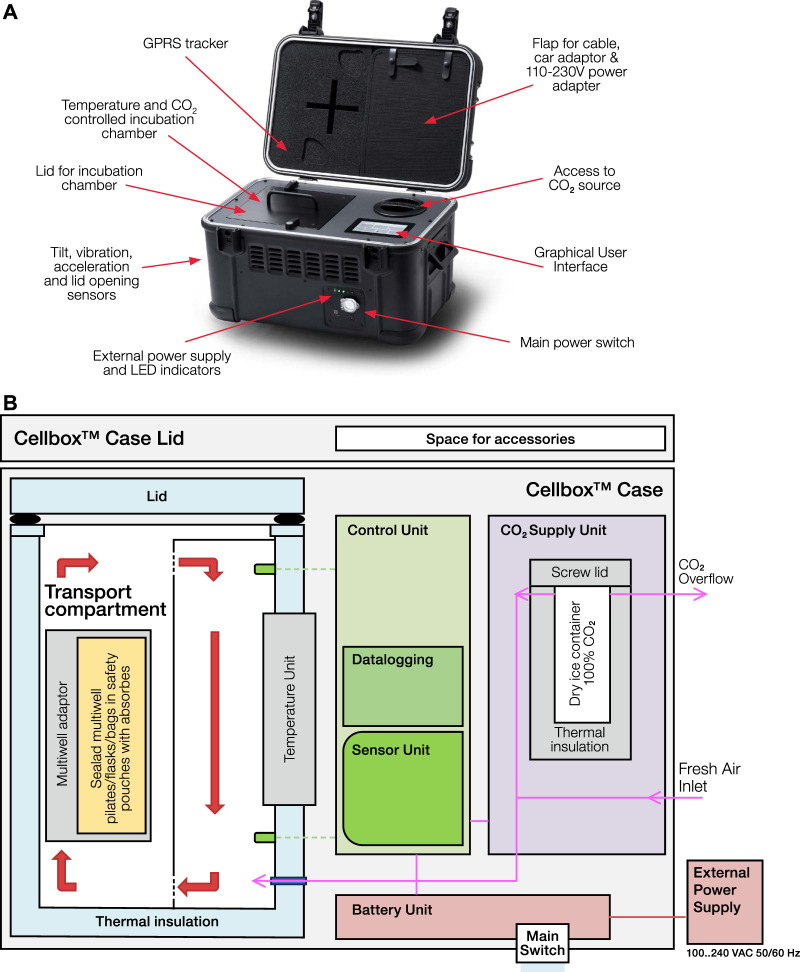
**(A)** The Cellbox™ system is a battery-powered, transportable CO_2_ incubator to ship biological material by road, sea, rail, and air. It features a robust outer case to withstand mechanical impact during transit and houses a lid-protected and temperature-controlled incubation chamber for various biological carriers. The Cellbox™ Shipper is additionally equipped with either a CO_2_ cartridge (Cellbox™ Shipper Ground) or a separate compartment filled with dry ice pellets (Cellbox™ Shipper Flight) that serves as a CO_2_ reservoir to control the CO_2_ concentration in the incubation chamber. The device is equipped with a display that serves as a graphical user interface, offering 21 CFR Part 11-compliant user management and workflows. A Bluetooth connection between a Cellbox™ system and smartphone allows the user to export monitored transport conditions, such as temperature and CO_2_ levels, and also mechanical transport data, such as tilt, acceleration, vibration, and lid sensor data, to allow for post-transport verification. The Cellbox™ system is rechargeable via an external power or car adapter, ensuring flexibility during operation. **(B)** Schematic overview of the Cellbox™ Shipper Flight. The Cellbox™ Shipper Flight comprises multiple functional units that jointly maintain defined transport conditions for cell cultures and ATMPs. The transport compartment is thermally insulated and designed to accommodate standard cell culture vessels. The sensor assembly and the axial fan, which ensure homogeneous temperature and gas distribution within the incubation compartment, are located behind a slotted separator. A temperature unit regulates the temperature in the transport area and maintains stable thermal conditions. The CO_2_ supply unit includes an insulated dry ice container, a valve, a pump, a sterile micro air filter, and a guaranteed controlled CO_2_ supply or fresh air into the transport compartment. In addition, it features a CO_2_ overflow that allows excess gas to vent to the environment without backpressure, as well as a fresh air inlet system that plays an important role in CO_2_ control during air transport. The control unit manages temperature and CO_2_ concentration, records operational data, and enables communication via an integrated user interface and Bluetooth. A separate sensor unit measures ambient pressure, internal temperature, acceleration, tilt, and vibration. Power is supplied by a rechargeable battery unit or an external 20 V DC source. The modular system design permits precise control of relevant parameters and continuous monitoring of transport conditions.

The Cellbox™ Shipper (see Figures 1A,B) is an electronic, battery-powered device designed to maintain precise temperature and CO_2_ control for the long-distance transportation of live cell-based materials and advanced therapy medicinal products (ATMPs). Suitable for flight or ground transport, the system ensures an accurate and uniform environment for sensitive biological samples housed in appropriate primary vessels (e.g., cell bags, well plates, and T-flasks). The Cellbox™ provides appropriate racks and holders to guarantee a safe transport of suspended or adherent cell types.

The Cellbox™ Shipper features a thermally insulated incubation chamber, which utilizes Peltier elements to maintain stable temperatures without a humidity control system. The system ensures pH and osmolality stability in the culture medium via a precise CO_2_ control mechanism. This is achieved through either dry ice sublimation for the Flight version or a pressurized CO_2_ cartridge for the Ground version. The version used in this experiment was the Cellbox™ Shipper Flight edition.

The device maintains a stable internal temperature selectable between 28 °C and 38 °C (here 37 °C), with homogeneity supported by circular airflow via internal fans. Gas concentrations are controlled and expressed as partial pressure of CO_2_, rather than percent volume, to better support the carbonate buffer equilibrium in the medium during flight transport. This independent measurement ensures stability regardless of ambient atmospheric pressure.

Designed for long-distance live cell shipments, the Cellbox™ Shipper Flight is fully compliant with European Agreement concerning the International Carriage of Dangerous Goods by Road (ADR) and International Air Transport Association (IATA) regulations by the International Civil Aviation Organization (ICAO) (ref. Technical Manual - Cellbox™ Shipper Flight (2.0) - v1.5 May 2024; Cellbox Solutions GmbH). Packaged in a polystyrene/carton overpack and accompanied by all necessary dangerous goods declarations, the device provides safe and regulated transport of up to 48 h of runtime while continuously monitoring and logging transport conditions.

Transport data, including gyro events, temperature, and CO_2_ levels, were logged throughout the journey and can be accessed via Bluetooth using dedicated mobile apps. A 21 CFR Part 11 software version is available upon request to fulfill the highest standard for data integrity, ensuring the highest standards for regulatory compliance.

For detailed information regarding the output parameters of the Cellbox™, see Supplementary Data 01, Figure 1.

### 3.2 Experimental setup

The flowcharts are mapped chronologically according to the practical implementation. Process steps are shown in blue, materials in orange, cultivation platforms in grey, and the associated quality control parameters in green. The preparatory NK cell isolations and expansions were a prerequisite for the subsequent bag test and shipment test phases (see Supplementary Data 02, Figure 1).

The bag test was carried out first in order to obtain a suitable bag type for the subsequent shipment test phase. A G-Rex^®^ 6-well plate with the same cell count and working volume used in the bags was used as a control. Only the bag type that showed the most matches compared to the G-Rex^®^ 6-well control was used for the further shipment test. All three test conditions were incubated at 37 °C and 5% CO_2_ for 3 days (see Supplementary Data 02, Figure 2).

**FIGURE 2:**
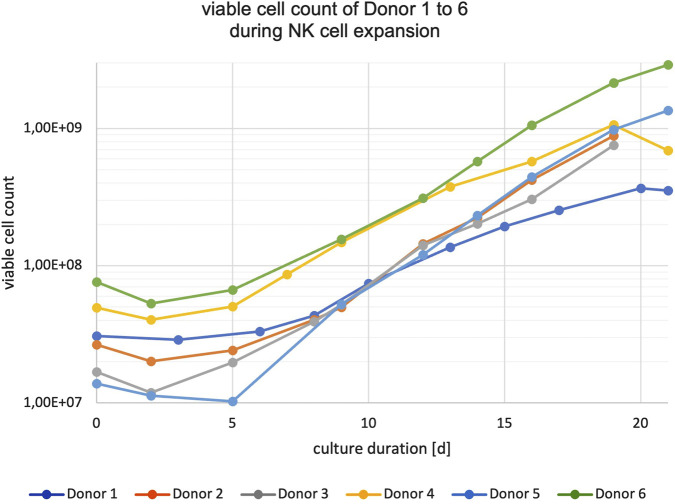
Growth curves of NK cell expansions for Donors 1 to 6 in logarithmic representation up to 21 days.

In the shipment test, two bags were filled with the same number of cells and working volume as the G-Rex^®^ 6 control. One bag and the G-Rex® control were incubated at 37 °C, 5% CO_2_ in a static incubator under optimal conditions. The other bag was placed in the Cellbox™ and subjected to real transport as described under “Shipment in the Cellbox™”. In addition, 14 vials with a working volume of 1 mL each (corresponding to the total cell number of the G-Rex® 6 control and the test conditions) were cryopreserved for the shipment test. After the shipment test, the NK cells from the bag of the Cellbox™ and the thawed NK cells from the cryovials were subjected to a 3-day recultivation phase (see Supplementary Data 02, Figure 3).

**FIGURE 3:**
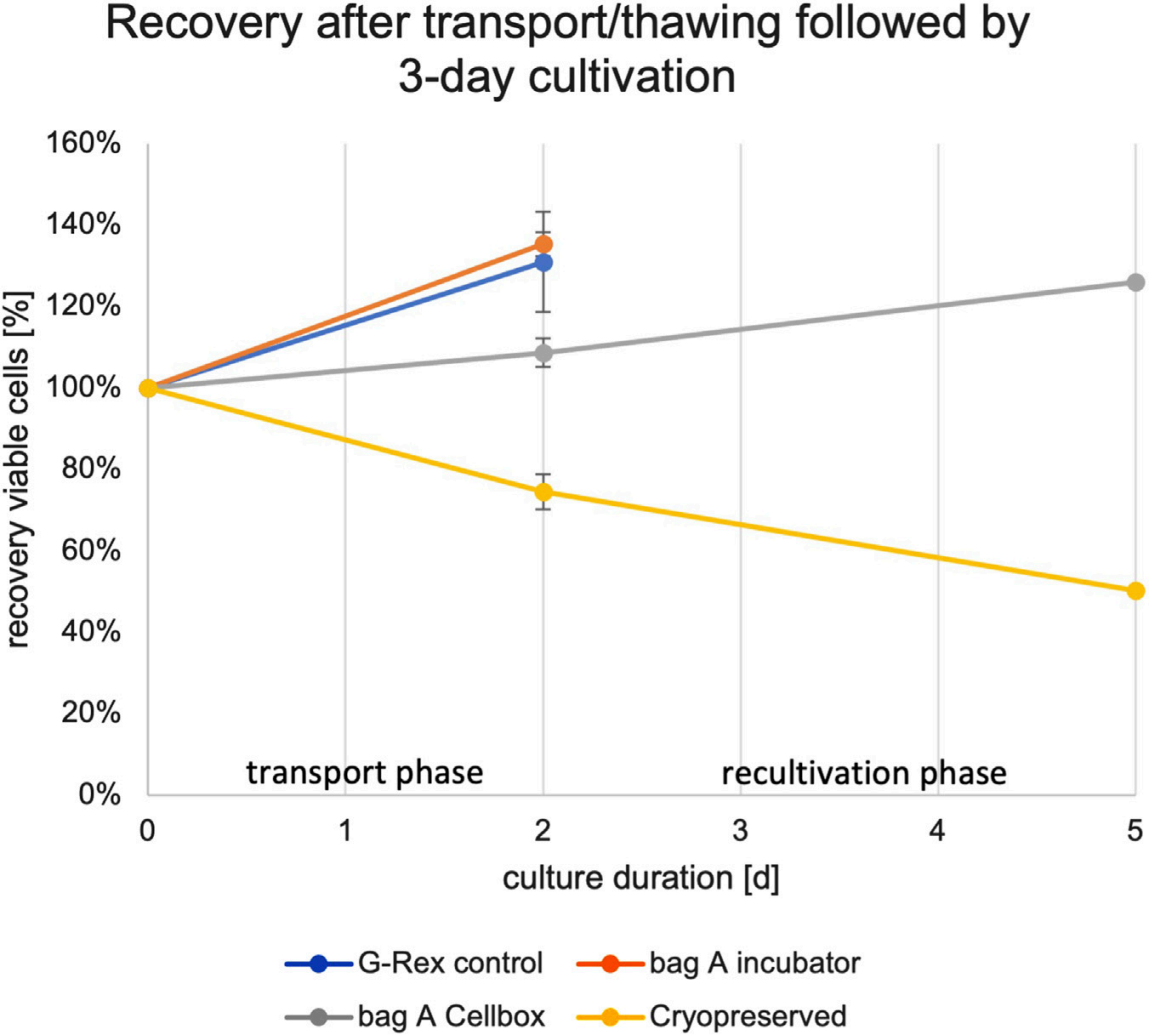
NK cell recovery after shipment (2 days) and recultivation phase (3 days) for G-Rex^*®*^ [control] and Bag A stored in a static incubator, Bag A stored and transported in the Cellbox™, and cyropreserved NK cells. The 100% value is based on the seeded viable cell count at the start of the shipment phase for Donors 5 and 6.

### 3.3 NK cell isolation

Six independent batches of NK cells were produced for this study. Following Ficoll-purification of PBMCs from donor buffy coats, NK cells were isolated via a negative immunomagnetic separation. Purification yields varied between 7.9% and 16.8% (defined by expression of CD56 and CD3), starting from PBMC counts comprised between 6.2 × 10^8^ and 1.1 × 10^9^ per buffy coat. Viabilities of purified NK cells measured ≥99% (99.5% ± 0.4%), and NK cell purity (defined by expression of CD56 and CD3) was shown to be >90% for all six donors directly after NK cell isolation (see Table 2).

**TABLE 2:** Viable cell counts of PBMC with appropriate yield, viability, and purity of isolated NK cells.

Donor	Viable PBMC count [cells]	Purification yield of NK cells per buffy coat [CD56^+^ & CD3^−^] [%]	Viability of isolated NK cells [%]	Purity of NK cells after NK cell isolation [CD56^+^ and CD3^−^] [%]
1	9.24 × 10^8^	7.9	99.5	94.6
2	7.31 × 10^8^	10.5	98.9	97.8
3	6.16 × 10^8^	11.0	99.2	93.1
4	8.71 × 10^8^	16.8	100.0	94.0
5	6.58 × 10^8^	8.6	100.0	93.1
6	1.07 × 10^9^	12.7	99.6	97.8

Detailed flow cytometry data prior to and after NK cell isolation can be found as an example for Donor 5 in the Supplementary Data 05, Table 1. The gating strategy prior to and after NK cell isolation can be found in the Supplementary Data 05, Figure 1 (A1 to A10 and B1 to B10).

### 3.4 NK cell expansion

NK cells were expanded without feeder cells in the G-Rex^®^ System from WilsonWolf (G-Rex^®^ 24-well and 6-well plates) using Miltenyi Biotec NK MACS^®^ media with 1% supplement, 5% human AB serum, IL-2 (500 U/mL), and IL-15 (140 U/mL) for up to 21 days. NK cell cultures began expanding after an initial lag phase of 5 days (8 days for Donor 1) (see Figure 2).

On average, NK cell cultures expanded between day 5 (or day 8 for Donor 1) until day 19 with an average doubling time of 3.1 ± 0.5 days (st dev) (data not shown). Cumulative population doublings varied between 3.5 and 6.6, corresponding to expansion folds of 12.0–97.7, respectively, for the Donor 1 and Donor 5 cultures (see Supplementary Data 03, Figure 1).

For all six cultures, robust CD56 expression was measured using flow cytometry, indicating high NK purity at the end of culture (detailed flow cytometry data after NK cell expansion—up to 19 days—is shown as an example for Donor 5 in the Supplementary Data 05, Tables 2, 3), while final cell populations were essentially devoid of CD3^+^, CD14^+^, and CD19^+^ events (see Supplementary Data 05, Table 2). Likewise, the expressions of a series of activating receptors that all serve as recognition receptors for the detection and elimination of transformed cells, that is, NKG2D, DNAM-1, NKp30, NKp44, and NKp46, were globally induced in NK cell cultures of all donors (see Supplementary Data 05, Table 3).

**TABLE 3:** Lactate concentration [mM] after 3 days of cultivation in the appropriate vessel for Donors 1–3.

Lactate [mM]	Bag test phase after 3 days		
Donor	G-Rex^®^ [control]	Bag A	Bag B
1	3.37	7.52	11.16
2	3.58	9.85	13.90
3	4.40	7.37	12.05
Average	3.78	8.25	12.37

The NK cells of all six donors showed different but NK-cell-typical glucose and lactate metabolism. A 75% media change was performed after a maximum of 4 days. During the lag phase of the NK cell cultivations (day 0–5 or day 8 for Donor 1), a low glucose consumption was to be expected, which, however, only increased to a limited extent even in later stages of the NK cell cultivation processes. However, the data did not allow for any conclusions regarding the expansion capacity of the NK cell culture to be drawn based on the glucose values (see Supplementary Data 04, Figure 1).

An inconclusive picture was also drawn regarding the NK cells’ ability to mediate cytotoxic activity against target cells using the calcein release assay, in which different activity patterns emerged in all six donors after the NK cell expansion phase. While Donor 1 and Donor 4 showed the lowest expansion rates, and Donor 5 (followed by Donor 6) showed the highest, we observed that the cytotoxic efficacies at lower E:T ratios (0.5:1 and 0.1:1) of the NK cells from Donor 1 and Donor 4 against K-562 cells were generally higher than those of Donors 5 and 6. Nevertheless, compared to NK cells from other donors, NK cells from Donors 5 and 6 potently mediated cell death in K-562 target cells, reaching maximal specific lysis values of more than 90% when high E:T ratios were used (E:T 5:1) (see Supplementary Data 06, Figure 1). In comparison to all the other donor NK cells, the cytotoxic performance of NK cells from Donors 5 and 6 fell short when lower E:T ratios were used (also see Supplementary Data 06, Figure 1). Furthermore, the cytotoxic activities of NK cell cultures from Donors 2 and 3 were consistent at both high and low E:T ratios, and the maximal specific lysis was >70% by an E:T ratio of 5:1 (also see Supplementary Data 06, Figure 1).

The NK cells from Donors 1–3 were used to test suitable transport bags. The cells from Donors 4–6, on the other hand, were used to test transport in the Cellbox™.

### 3.5 Transferring NK cells from G-Rex^®^ to cell culture bags does not alter their biology or function

Before addressing the question of the transport of the cells, either in culture or cryopreserved, we wanted to find the best container to ensure full containment, lack of contamination, and preservation of quality and function of the NK cells while being shipped (see Supplementary Data 01, Figure 2). Three independent donor-derived NK cell cultures (Donors 1–3) were used to select the optimal cell culture bag to store and transport NK cells in the Cellbox™. Cells were seeded at 1 × 10^7^ NK cells/mL in growth media, and 35 mL of cell suspension were dispatched either in a G-Rex^®^ 6-well (as reference condition), OriGen PermaLife™ Cell Culture Bag PL70-2G (Bag A), or Miltenyi Biotec MACS^®^ GMP Cell Differentiation Bag - 100 (Bag B). The two gas-permeable cell bags included in the study were chosen based on their respective lower and higher CO_2_ permeability, that is, 0.33 mL CO_2_/(cm^2^ × day) versus 2.6 mL CO_2_/(cm^2^ × day), respectively. Incubation of the three subcultures was performed in the static incubator at 37 °C and 5% CO_2_, and cells were assessed after 24 h and 72 h of culture for cell count, viability, phenotype, exhaustion markers, K-562 killing activity, metabolite concentrations, and pH in media. Microscopical observations of the cell cultures were performed as well.

Although cell proliferation was observed within the cell bags, cell growth was lowest in Bag B (x-fold in average: 1.52) compared to the G-Rex^®^ control (x-fold in average: 1.81; data not shown). The main difference between the performance of the two bags was measured at the level of metabolite concentrations after 3 days of cultivation. In Bag B, the LAC concentration reached a mean value of 12.4 mM compared to 3.8 mM in the G-Rex^®^ and 8.3 mM in Bag A (see Table 3).

Corresponding to the LAC accumulation, the GLC consumption was higher in Bag B (Supplementary Data 04, Figure 2).

After the 3-day cultivation during the bag test phase, the NK cells of each condition were screened by flow cytometry, and the G-Rex^®^ conditions were used as a control. Overall, most of the determined markers show stable characteristics for both bag types compared to the G-Rex^®^ conditions (see Supplementary Data 05, Table 4). The most striking differences in surface receptor expression patterns were shown in the NK cells cultured in Bag B compared to those cultured in G-Rex^®^, when NKG2D values were examined. NKG2D expression was reduced in NK cells that were cultured in Bag B compared to the control. Furthermore, the expression of CD56+ PD-1+ for Donor 1 showed higher levels in Bag A (13.96%) and Bag B (9.32%) conditions. For Donor 2, the control also showed an unusually high level of CD56+ PD-1+ (30.99%). In Bag B for Donor 3, higher CD56+ PD-1+ levels than the control were observed (see Supplementary Data 05, Table 4). The level of surface receptor expression and the cytotoxic functionality of the Bag A NK cell cultures were found to more closely resemble the control NK cell cultures when compared with Bag B cultures (see Supplementary Data 05, Table 4; Supplementary Data 06, Figure 2).

**TABLE 4:** Rating points for evaluating the suitability of the bags.

Rating points
5 = very good
4 = good
3 = Ok
2 = acceptable
1 = not acceptable

Altogether, an activation of NK cell metabolism was observed as cells were transferred into cell culture bags (see Supplementary Data 04, Figure 2). When cell killing activity was assessed, NK cells cultured in bags showed higher cytotoxic activity against K-562 cells compared to cells incubated in G-Rex^®^ for 3 days (Supplementary Data 06, Figure 2). No statistically significant difference between Bag A and Bag B cultured cells could be observed in these experiments using a calcein release assay and K-562 as target cells (data not shown).

The decision to use Bag A for further assessment in the Cellbox™ was finally based on the following rating point table (see Table 4) in combination with the evaluated benchmark table in Table 3.

The optimum score of five points was attained when the parameter result was comparable to the control (see Table 5, parameter “doubling time”). If the results of one bag type were superior for a particular parameter, that bag type was given five points, while the control was given fewer points than the optimum (see Table 5 parameter for the “pH value”).

**TABLE 5:** Summary of benchmark evaluation of bag suitability.

Parameter	G-Rex^®^ control	Bag A	Bag B
Type	6-well	PermaLife™ Cell Culture Bag	MACS^®^ GMP Cell Differentiation Bag - 100
Cat. No.	80240M	PL70-2G	170-076-400
pH value	Relatively high	Lower than control	Lowest
Viable cell count	Increased	Increased	Increased
Doubling time	As usual	Similar to control	Similar to control
Viability	Constant high	Constant high	Constant high
Morphology	Biggest cell cluster	Small cell cluster, but a homogenized cell layer	Small cell cluster, but a homogenized cell layer
GLC Metabolism	Highest concentration	Middle	Lowest concentration
LAC Metabolism	Lowest concentration	Middle	Highest concentration
Calcein activity	As usual	Slightly higher than control	Slightly higher than control
Flow cytometry	As usual	Comparison to control at day 22/bag day 3	Comparison to control at day 22/bag day 3
		Similar to control	Similar to control
Handling	As usual	OK, slightly easier than Bag B	Ok
Used volume	As usual	Good	35 mL is not enough for this bag type
Usable for cryopreservation	No	Yes	No
Price for cultivation of 35 mL	17 € (99 € per 6-well plate)	56 € (per bag)	41 € (206 € per pack)
Sum rating points	57	54	47

The three most prominent decision criteria were the following:

1. The gas permeability, which was higher for the Fluoro-ethyl polymer membrane in Bag A.
2. Differences in lactate accumulation and glucose consumption, which were heightened in Bag B.
3. Working volume usability of Bag B, where 35 mL was seemingly too low.

Bag A received a rating of 54 points, compared to 47 points for Bag B. Therefore, Bag A was chosen for further transport testing.

### 3.6 Shipping NK cells under optimal cell culture conditions maintains their counts, viability, and metabolic profile

After the Cellbox™ arrived, the bags were checked externally for leaks. This revealed that the bag from Donor 4 had a reduced volume (25 mL instead of 35 mL). This was caused by a leak at the bag nozzle. This was not observed for Donors 5 and 6. The viable cell count in the Cellbox™ was reduced for Donor 4 due to the leakage (82%, from 1.69 × 10^8^ NK cells to 1.39 × 10^8^ NK cells), whereas Donor 5 (106%) and Donor 6 (111%) showed an increase in viable cell count compared to cell counts obtained before shipping started. In comparison, the viable cell counts of the G-Rex^®^ 6 control (the average of all donors was 123% ± 17%) and in Bag A in the incubator (125% ± 19%) also increased for all three donors. The viable cell count of the cryopreserved cells decreased to 78% ± 7% after thawing (see Supplementary Data 03, Table 1).

None of the tested conditions affected cell viability, which remained above 95% on average, even for cryopreserved cells. The lowest cell viability was 96% for Donor 4 immediately after thawing (see Supplementary Data 03, Table 2).

The pH value varied only minimally between the three ambient test conditions (control, Bag A in an incubator, and Bag A in Cellbox™) and was between 7.3 and 7.5 for Donors 4–6.

With regard to the metabolic profile for glucose and lactate, the results from the bag test were confirmed. The NK cells that were cultured in a G-Rex^®^ 6-well plate (control) showed the lowest consumption of glucose at the highest cell expansion compared to the other test conditions analyzed (see Supplementary Data 04, Figure 3A, B). Correspondingly, the lactate concentration was lowest there at 5.03 mM for Donor 5. An increased consumption of glucose (16.05 mM–12.45 mM) and thus an increase in lactate (0.04 mM–7.29 mM) was measured in media from Bag A that was placed in the stationary incubator. The expansion of the NK cells was lower in Bag A (1.67 × 10^8^ NK cells) than the G-Rex® control (1.75 × 10^8^ NK cells). A higher consumption of glucose (16.05 mM–11.34 mM) was observed in Bag A, transported in the Cellbox™, compared to the bag cultivated using the stationary incubator. Accordingly, the lactate concentration in Bag A increased to a maximum of 11.34 mM with comparatively low final NK cell numbers of 1.33 × 10^8^ cells (see Table 6).

**TABLE 6:** Culture parameters in G-Rex control in an incubator, bag control in an incubator, and bag in a Cellbox^TM^ system after 2 days for Donor 5.

Parameter	Incubator control	Incubator Bag A	Cellbox™ with Bag A
Viable cell count	1.75 × 10^8^	1.67 × 10^8^	1.33 × 10^8^
Viability [%]	99.3	98.2	96.3
pH value	7.5	7.3	7.4
Glucose [mmol/L]	13.62	12.45	11.34
Lactate [mmol/L]	5.03	7.29	10.18

With regard to the flow cytometry profile, results for NK cells cultured under “live” test conditions were comparable to those obtained during the bag test. Surface marker expression of CD56+ DNAM1+ was increased in cells that were cultured in bags compared to the control cultured cells. Increased expression of CD56+ NKp44+ was observed in both bags (77% each) compared to the G-Rex^®^ control (54%) (see Supplementary Data 05, Table 5). In contrast, all other markers showed only slight deviations from the control (see Supplementary Data 05, Table 5).

With regard to the cytotoxic activity under the “live” conditions, the NK cells from Donors 4–6 showed different results. NK cells from Donor 4 mediated a maximal specific lysis of 95% (E:T 5:1) for the test condition (incubator with Bag A), and the effect was shown to be E:T ratio-dependent (50% max. Specific lysis when an E:T ratio of 0.1:1 was used) (see Supplementary Data 06, Figure 4A). The control (G-Rex^®^ 6) showed a relative toxicity of 87% at an E:T 5:1 and also achieved a relative toxicity of 50%. The NK cells from the Cellbox™ showed toxicities of max. 76% (E:T 5:1) and min. 48% (E:T 0.1:1) (see Supplementary Data 06, Figure 3). NK cells that were isolated from Donor 5 showed comparable cytotoxic activity independent of the culture conditions (E:T 5:1: 72%–79%; E:T 0.1:1: 14%–16%) (see Supplementary Data 06, Figure 3). NK cells from Donor 6 showed increased cytotoxic activity against K-562 cells when cultured in bags and showed slight differences between Bag A cultured in the Cellbox™ and Bag A cultured in a static incubator (both conditions: E:T 5:1: 80%; E:T 0.1:1: 19%) (see Supplementary Data 06, Figure 3).

For Donor 4, the comparison of NK cells regarding their cytotoxic potential was made between those cultivated in Bag A within the Cellbox™ and cryopreserved and thawed NK cells. At an E:T ratio of 5:1, both conditions showed nearly the same killing efficacy (76% for NK cells in Bag A within the Cellbox™, and 74% for thawed NK cells). At a reduced E:T ratio of 1:1, NK cells transported in the Cellbox™ demonstrated a slightly higher killing efficacy than thawed NK cells (79% for NK cells in Bag A within the Cellbox™, and 73% for thawed NK cells). At an E:T ratio of 0.5:1, a greater difference was observed between the two conditions (71% for NK cells in Bag A within the Cellbox™, and 51% for thawed NK cells). This effect was more pronounced at the lowest E:T ratio of 0.1:1 (48% for NK cells in Bag A within the Cellbox™, and 18% for thawed NK cells) (see Supplementary Data 06, Figure 4A).

At reception after shipment, microbacterial safety testing was performed for the three Cellbox™ “live” shipments, and NK cell products were shown to be sterile and *Mycoplasma* free. Endotoxin levels were below 0.05 EU/mL.

### 3.7 Recultivation of NK cells under optimal cell culture conditions in the G-Rex^®^ formate

For a deeper investigation of possible long-term effects of the transport at 37 °C or cryostorage, and also because it is known that thawed NK cells must recover in the presence of IL-2 to retrieve their cytotoxic capabilities (Berg et al., 2009), a 3-day recultivation period was performed for frozen cells and cells shipped in the Cellbox™.

The calculation of cell counts for the transport phase and subsequent recultivation was performed with NK cells from Donors 5 and 6 (due to leakage during transport of NK cell cultures from Donor 4, these data were not obtained).

Cells transported in the Cellbox™ did slightly grow or maintain their numbers with 126% of average recovery after the recultivation phase for Donors 5 and 6 (see Supplementary Data 03, Table 4; Figure 2 below). In contrast, thawed cells showed a sharp decrease in NK cell numbers to 71% (Donor 5) and 78% (Donor 6) during the transport phase. A further decrease during the recultivation phase compared to the initial amounts of cells seeded was identified (50% for Donor five and 51% for Donor 6) (see Supplementary Data 03, Table 4, and Figure 3 below).

Such a result could be related to a phenomenon called cryopreservation-induced delayed onset cell death (CIDOCD), where cells that have suffered irreversible damage from cryopreservation are not detected as dead cells on thawing, but eventually die over the next days (Baust et al., 2001).

After 3 days in culture, apoptotic cells have died, and a healthy culture has likely resumed, as indicated by high viabilities of 98% in both conditions (Bag A shipped in Cellbox™ and cryopreserved NK cells) after recultivation for Donors 5 and 6 (see Supplementary Data 03, Table 5). Regarding the phenotype and activity assessment, cryopreserved/thawed and 3-day-cultivated NK cells showed a receptor profile comparable to the NK cells transported in Bag A and the Cellbox™. The NKG2D^+^ receptor was shown to be highly expressed on the cryopreserved NK cells (prior recultivation: 83%, after recultivation: 71%) compared to Cellbox™ transported NK cells (Bag A). These cells showed a comparatively stable level of NKG2D expression over the 3-day culture period (prior recultivation: 53%, after recultivation: 59%) (see Supplementary Data 05, Table 6). The total impact of cryopreservation on cell counts was determined by adding the cell losses measured after thawing and after recultivation, which together amount to nearly 50% (on average, 50% ± 1% st dev). In contrast to the severe cell loss caused by cryopreservation, the same calculation shows that shipping primary NK cells in the Cellbox™ resulted in a recovery of 126% ± 0% of the transported cells (see Supplementary Data 03, Table 4).

The cryopreserved and thawed NK cells generally showed lower levels of the following surface markers directly after thawing compared to the NK cells in Bag A transported within the Cellbox™ (see Supplementary Data 05, Table 6, especially for Donor 4):

- Immune status: CD4^+^, CD16^+^ CD56^+^, CD45^+^.
- Receptor: CD56^+^, CD56 high, CD56^+^ CD16^+^, CD56^+^ DNAM1^+^, CD56^+^ NKp30^+^, CD56^+^ NKp44^+^, CD56^+^ NKp46^+^.
- Exhaustion: CD56^+^ CD16^+^, CD56^+^ Tim3^+^.

Directly after thawing, cryopreserved NK cells showed elevated expression intensities for CD56 dim and NKG2D^+^ as described above. After subsequent recultivation in G-Rex^®^ 24 format, all markers recovered almost completely and corresponded with the surface marker profile of the NK cells from the Cellbox™. For the NK cells that were transported in the Cellbox™, no relevant changes were observed in the surface marker profile at the beginning and end of recultivation in the G-Rex^®^ 24 format (see Supplementary Data 05, Table 6).

To further elaborate on the quality of “live-shipped” and cryopreserved cells, expression levels of a series of activating NK receptors were analyzed and compared to the standard expression levels on cells cultured in G-Rex^®^ in the static incubator. We found very similar expression profiles when comparing NK cells shipped in the Cellbox™ and cells incubated in the static incubator, either in G-Rex^®^ or in Bag A (see Supplementary Data 05, Table 6, especially for Donor 4). In contrast, cryopreserved NK cells had a distinct expression profile of most of the markers tested (see above).

NK cell cytotoxicity was analyzed post-shipment or cryopreservation by the same calcein release assay using K-562 target cells as used at the end of the expansion.

Immediately after thawing the cryopreserved NK cells, their activity was observed to be lower at E:T ratios of 1:1, 0.5:1, and 0.1:1 compared to NK cells transported using the Cellbox™. These differences were not prominent when an E:T ratio of 5:1 was used, with only slight differences noted compared to the cells transported with the Cellbox™ (see Supplementary Data 06, Figure 4A).

After the 3-day recultivation phase, both the cryopreserved and recultivated NK cells and the Cellbox™-transported NK cells showed higher activity at an E:T ratio of 5:1 compared to the beginning of the recultivation phase. In contrast, for cryopreserved NK cells, no changes in cytotoxic activity were detected for any of the other E:T ratios analyzed (see Supplementary Data 06, Figures 4A, B). NK cells transported in the Cellbox™ showed a slight decrease in cytotoxicity at the end of the recultivation phase by an E:T ratio of 0.1:1 (27%) in comparison to the beginning of the recultivation (48%) (see Supplementary Data 06, Figures 4A, B).

## 4 Discussion

The use of DMSO in the development of cell and gene therapy products has grown historically, and despite its toxic properties, scientists and physicians have few clinical alternatives for storing and shipping cells (Awan et al., 2020). In the first place, cryopreservation imposed itself over the “just-in-time” manufacture and short-term storage to become a standard in autologous cell therapy manufacturing as it stabilizes the product while allowing time for quality control and release activities. In addition, it provides a buffer for shipment delays, which are frequent when drug products must cross borders or oceans from a central manufacturing facility to other countries or continents. It also allows the medical team to fully control the timing of administration. Finally, it is conditional on the manufacturing of larger batches of product, either for therapies needing repeated dosing or for allogeneic products that can be prepared in larger amounts at lower cost and made available for subsequent use at any time with consistent quality (Hopewell et al., 2024).

However, for cryopreservation to be used with optimal success, it is critical that the entire protocol of freezing, storage, and thawing procedures is adequately and specifically developed for the product at stake, then carried out in the precisely prescribed manner by both the manufacturing and clinical team (Hawkins et al., 2017; Woods et al., 2016). Today, intracellular-like solutions (CryoStor^®^ and UnisolTM) have become the gold standard DMSO-based cryoprotectants for therapeutic products (Mathew et al., 2002; Baust et al., 2002; Stylianou et al., 2006; Baust, 2005; Clarke et al., 2009). Through their multi-component formulation, they modulate the stress response resulting from the freeze–thaw process and reduce the number of cells that will perish by necrosis, programmed apoptosis, and secondary necrotic cell death, hours to days after thawing in a phenomenon known as cryopreservation-induced delayed onset cell death (CIDOCD) (Baust et al., 2001). Still, continued cell loss (ranging from 10 to >50% depending on the cell type), compromised function in complex cells and tissues, spontaneous differentiation in native stem cell populations, and dependence on DMSO remain major challenges for numerous therapeutic products (Woods et al., 2016; Baust et al., 2002). These drawbacks have prompted research into alternatives, ranging from the development of new biocompatible cryoprotectants to optimization strategies involving post-thawing recovery cultures to increase the *in vivo* activity of therapeutic cells to options for shipping viable cells at ambient temperature, usually by embedding them in gels or matrices. (Heydarzadeh et al., 2022; Cottle et al., 2022). These developments are certainly worthy and encouraging, although not applicable yet at a large or industrial scale. Our study contributes to these efforts by providing a proof-of-concept that a transportable incubator could be used instead of cryogenic storage to safely transport natural killer cells, where cellular metabolism outside of dedicated cell culture facilities under normothermic and isocapnic conditions is maintained. We chose to address the challenge of NK cells specifically, as on the one hand, they are highly regarded drug candidates in immuno-oncology, and on the other hand, serious doubts have been raised about their capability to recover their full therapeutic potential after thawing.

This study confirms that cryopreservation has a major negative impact on the quality of expanded and *ex vivo*-activated NK cells. We have methodically characterized six independent cultures of NK cells. Three of these NK cell cultures were extensively analyzed in terms of their quality attributes and performance when either cryopreserved and thawed or shipped “live” in a Cellbox™ or incubated for 2 days in a static incubator. Cryopreservation was performed by using CryoStor^®^ CS10 solution containing 10% DMSO. It is worth mentioning that achieving such a high recovery of living NK cells post-cryopreservation in a reproducible manner is due to an optimized protocol using a controlled rate freezer with a gradient of temperature, controlled by a sample probe, and specific care at thawing to avoid excessive DMSO toxicity and osmotic shock. Thawing was done manually, five vials at a time, with high attention to limit osmotic stress by small step dilution and sheer stress control. Optimization of manual handling steps resulted in an average of 78% (see Supplementary Data 03, Table 1) cell count recovery with viabilities that remained higher than 96% (see Supplementary Data 03, Table 2). In spite of optimization and good results at thawing, approximately 1/3 of the thawed cells died during the 3-day recultivation period (see Supplementary Data 03, Table 3; Figure 2). Such a result is consistent with the fact that cells that have suffered irreversible damage from cryopreservation are not detected as dead cells at thawing but will eventually dye in the next couple of days from CIDOCD.

The flow cytometry data of the cryopreserved NK cells showed reduced expressions of the characteristic NK cell markers, CD56, and other activation receptors, including CD16, DNAM-1, and NCR1-3 (see Supplementary Data 05, Tables 5, 6). The flow cytometry receptor profile of the NK cells transported within the Cellbox™ showed, for most of the receptor expressions, a comparable profile to the G-Rex^®^ control condition during transport and recultivation phase (identified differences were described above in Sections 6 and 7). Similar loss of functional receptor expression on clinical-grade freshly thawed NK cells was reported by others (Berg et al., 2009), which in turn, did correlate with a loss of cytotoxic activity (Lapteva et al., 2012; Damodharan et al., 2020; Mark et al., 2020; Berg et al., 2009). We found that cryopreserved NK cell products were less active against K-562 target cells than their respective counterparts incubated in G-Rex^®^ for 2 days.

As shown by others, the restoration of receptor expression and cytotoxic activity of cryopreserved NK cells after a recultivation phase appears to be possible (Mata et al., 2014). Unfortunately, in clinical practice, frozen cells are not recultivated before injection into the patient but are administered immediately. Whether this recovery of activity also occurs *in vivo*, that is, after injection into the patient, is unclear. There is clear evidence that infusing fresh NK cells is critical to their expansion *in vivo* and that, without a recovery period, cryopreserved NK cells do not expand or persist *in vivo* (Miller et al., 2014; Szmania et al., 2015). Thawed NK cells, therefore, would not deliver their full therapeutic potential.

In contrast, NK cell products shipped for 2 days in a gas-permeable cell bag in the Cellbox™ maintained their numbers, viability, ability to proliferate, phenotype, and cytotoxic activity. The feasibility of a live cell shipment was shown, as the integrity of the cell bag was checked upon Cellbox™ arrival at the destination. The sterility of the cell products was not compromised by the use of the Cellbox™; endotoxins were below the clinically acceptable threshold, and *Mycoplasma* was undetectable when the culture bag was intact. These are all sine *qua non* safety conditions to further evaluate the Cellbox™ for clinical applications.

The development of Cellbox™-based transportation can be seen as an innovative alternative to cryopreservation and could possibly synergize with options reviewed by Heydarzadeh et al. (2022) and Cottle et al. (2022) that would simplify normothermic shipments. However, there are gaps to be filled for seamless implementation in the clinics. Cellbox™ might be the missing tool to guarantee controlled shipment for many cell-based regenerative medicine therapy candidates that do not have the option of cryopreservation (for example, retinal or cardiac muscle patches, engineered beta cell islets) and for which regulatory approval has been obtained for preparation of the product at the hospital by manual and open steps.

For other cell-based products, as for the NK cells in this study, it is likely that the media used to ship the cells must be changed and washed before administration to the patient. The final formulation step will have to be set up at the clinical site. Closed and automated solutions already exist that would streamline final formulation at the hospital pharmacy while ensuring safety and the lack of contamination.

To conclude, great efforts must be made to take logistics into account in the development of cell-based product manufacturing. Cryopreservation is not necessarily the best or unique solution for all cell-based therapies and tissue-engineered products, especially if the quality and efficacy of these products rely on more sophisticated handling or manufacturing protocols. Temperature and gas-controlled transportable incubators like the Cellbox™ have a role to play in distribution chains that support the transport of live cells and engineered tissues for the dissemination to patients.

## Data Availability

The original contributions presented in the study are included in the article/Supplementary Material; further inquiries can be directed to the corresponding authors.
