# Supplementary material for: “Live” cell shipment—a forward-looking transport option for cryo-sensitive cell-based therapies
Source: Front Bioeng Biotechnol. 2025 Dec 9;13:1706927. doi: 10.3389/fbioe.2025.1706927 (PMC12723144; doi:10.3389/fbioe.2025.1706927)
Supplement: Supplementary file 1 [file Presentation1.pptx]

## Slide 1
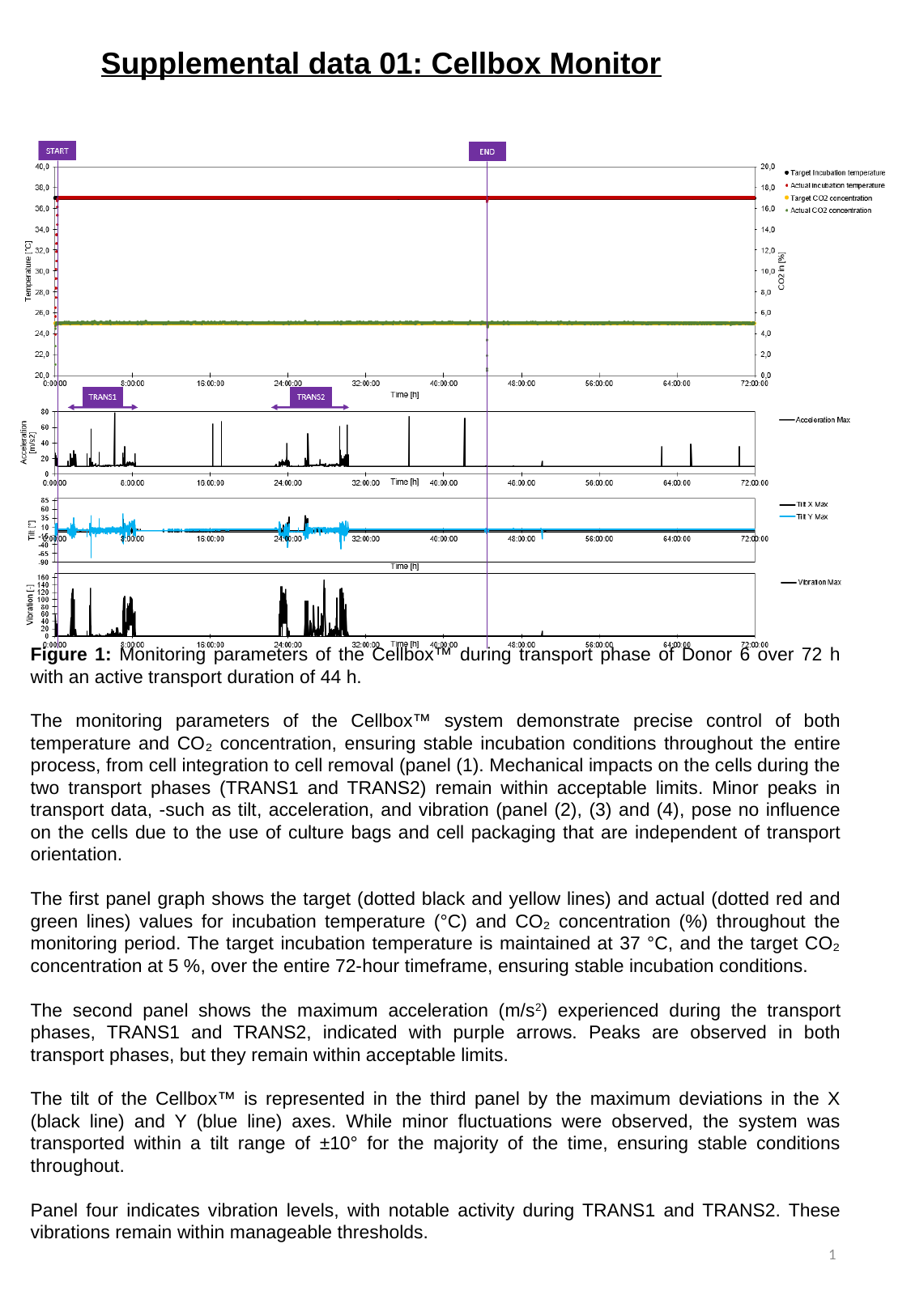

Supplemental data 01: Cellbox Monitor
Figure 1: Monitoring parameters of the Cellbox™ during transport phase of Donor 6 over 72 h with an active transport duration of 44 h.
The monitoring parameters of the Cellbox™ system demonstrate precise control of both temperature and CO₂ concentration, ensuring stable incubation conditions throughout the entire process, from cell integration to cell removal (panel (1). Mechanical impacts on the cells during the two transport phases (TRANS1 and TRANS2) remain within acceptable limits. Minor peaks in transport data, -such as tilt, acceleration, and vibration (panel (2), (3) and (4), pose no influence on the cells due to the use of culture bags and cell packaging that are independent of transport orientation.
The first panel graph shows the target (dotted black and yellow lines) and actual (dotted red and green lines) values for incubation temperature (°C) and CO₂ concentration (%) throughout the monitoring period. The target incubation temperature is maintained at 37 °C, and the target CO₂ concentration at 5 %, over the entire 72-hour timeframe, ensuring stable incubation conditions.
The second panel shows the maximum acceleration (m/s2) experienced during the transport phases, TRANS1 and TRANS2, indicated with purple arrows. Peaks are observed in both transport phases, but they remain within acceptable limits.
The tilt of the Cellbox™ is represented in the third panel by the maximum deviations in the X (black line) and Y (blue line) axes. While minor fluctuations were observed, the system was transported within a tilt range of ±10° for the majority of the time, ensuring stable conditions throughout.
Panel four indicates vibration levels, with notable activity during TRANS1 and TRANS2. These vibrations remain within manageable thresholds.
1
