# Supplementary material for: “Live” cell shipment—a forward-looking transport option for cryo-sensitive cell-based therapies
Source: Front Bioeng Biotechnol. 2025 Dec 9;13:1706927. doi: 10.3389/fbioe.2025.1706927 (PMC12723144; doi:10.3389/fbioe.2025.1706927)
Supplement: Supplementary file 2 [file Presentation4.pptx]

## Slide 1
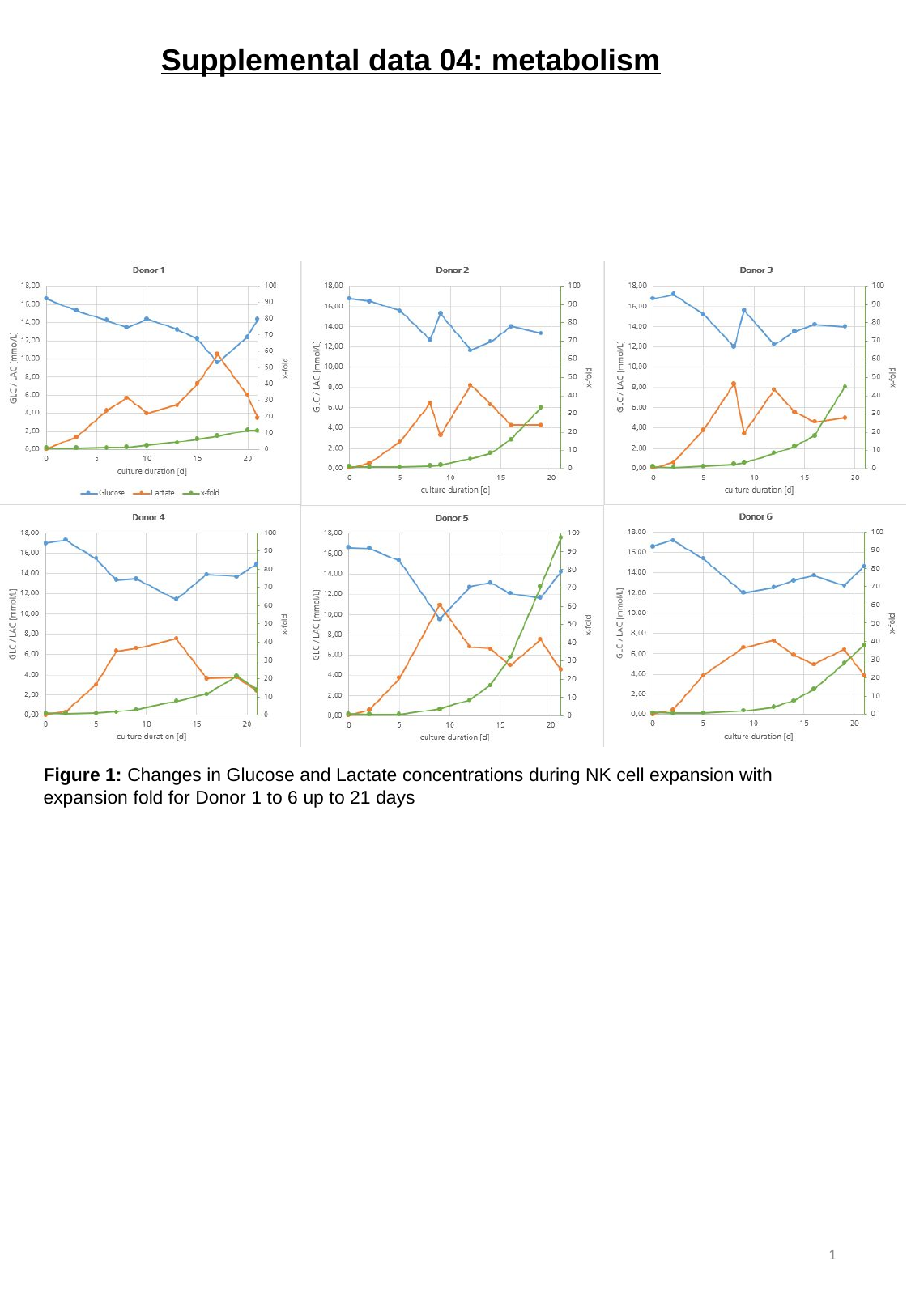

Supplemental data 04: metabolism
Figure 1: Changes in Glucose and Lactate concentrations during NK cell expansion with expansion fold for Donor 1 to 6 up to 21 days
1

## Slide 2
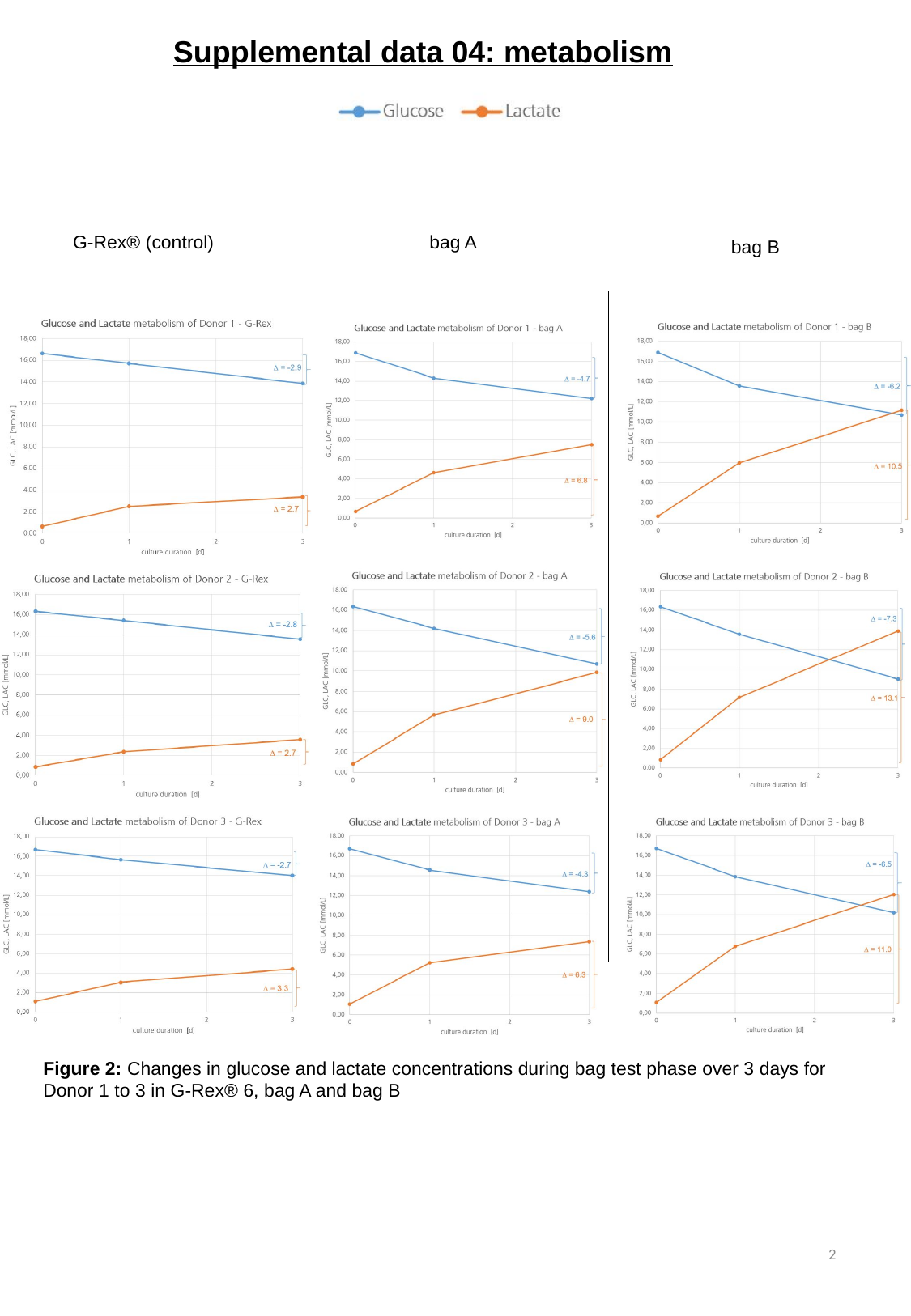

Supplemental data 04: metabolism
G-Rex® (control)
bag A
bag B
Figure 2: Changes in glucose and lactate concentrations during bag test phase over 3 days for Donor 1 to 3 in G-Rex® 6, bag A and bag B
2

## Slide 3
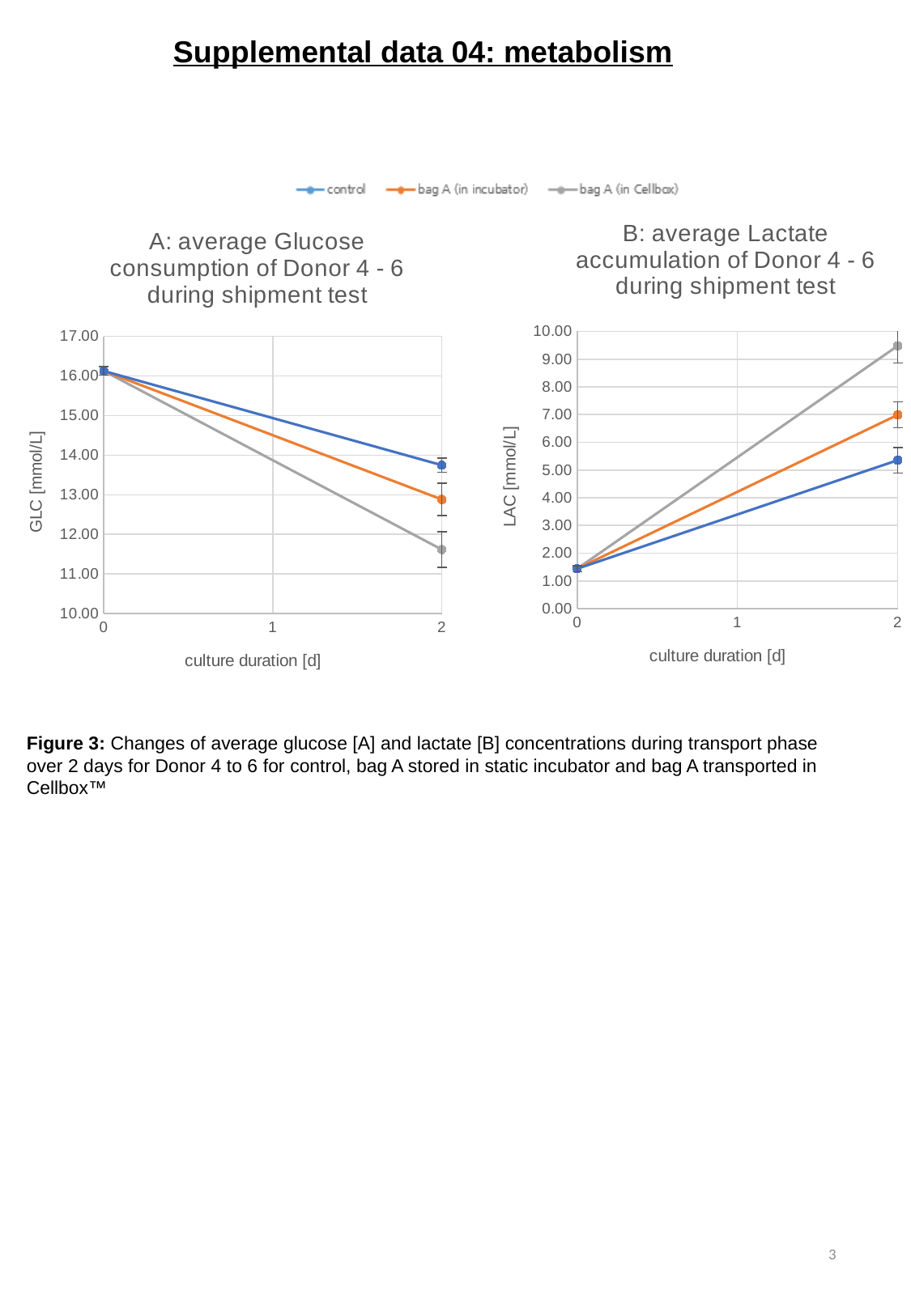

Supplemental data 04: metabolism
### Chart: B: average Lactate accumulation of Donor 4 - 6 during shipment test
| Category | | | |
|---|---|---|---|
### Chart: A: average Glucose consumption of Donor 4 - 6 during shipment test
| Category | | | |
|---|---|---|---|Figure 3: Changes of average glucose [A] and lactate [B] concentrations during transport phase over 2 days for Donor 4 to 6 for control, bag A stored in static incubator and bag A transported in Cellbox™
3
