# Supplementary material for: “Live” cell shipment—a forward-looking transport option for cryo-sensitive cell-based therapies
Source: Front Bioeng Biotechnol. 2025 Dec 9;13:1706927. doi: 10.3389/fbioe.2025.1706927 (PMC12723144; doi:10.3389/fbioe.2025.1706927)
Supplement: Supplementary file 3 [file Presentation6.pptx]

## Slide 1
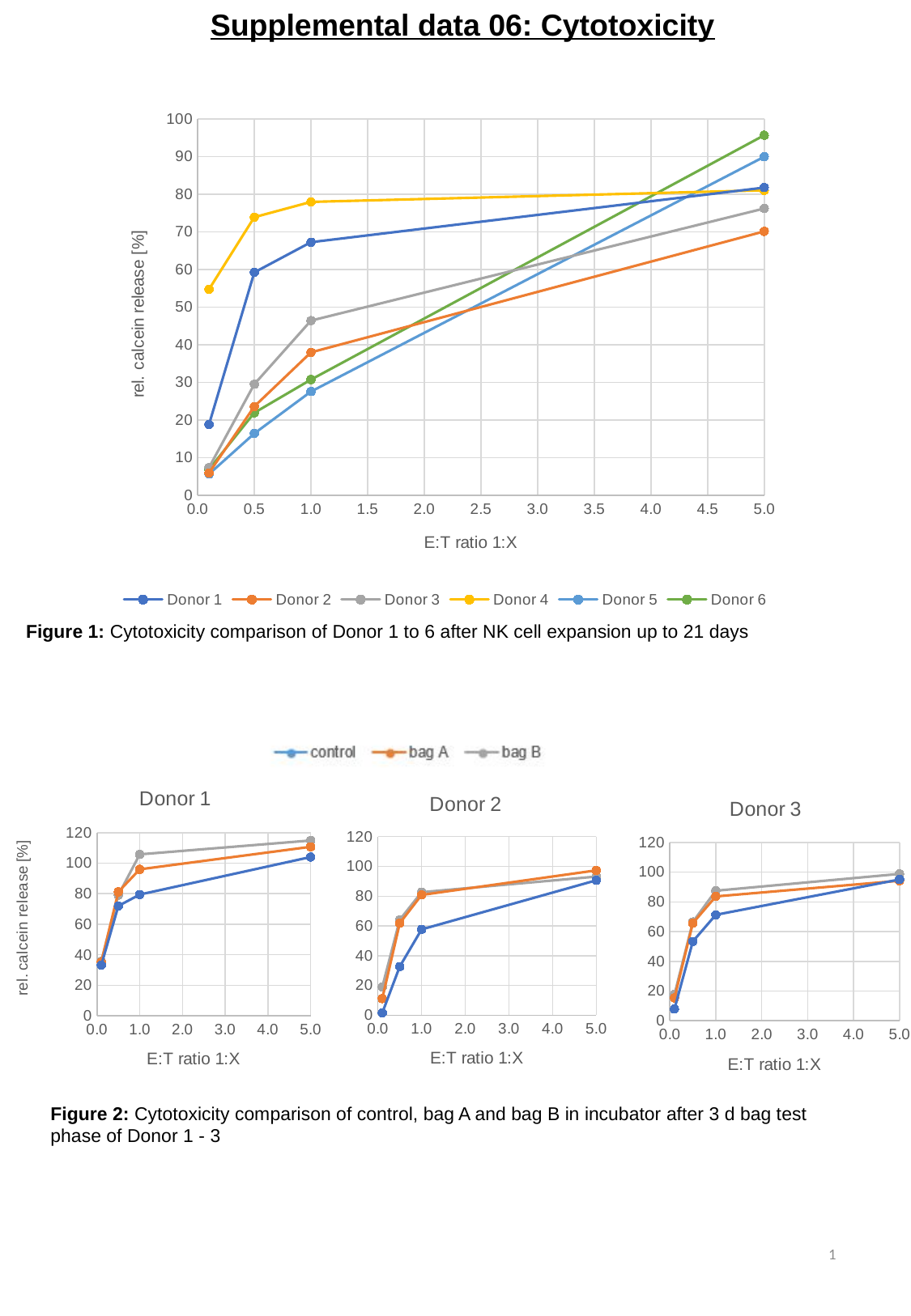

Supplemental data 06: Cytotoxicity
### Chart
| Category | | | | | | |
|---|---|---|---|---|---|---|Figure 1: Cytotoxicity comparison of Donor 1 to 6 after NK cell expansion up to 21 days
### Chart: Donor 1
| Category | | | |
|---|---|---|---|
### Chart: Donor 2
| Category | | | |
|---|---|---|---|
### Chart: Donor 3
| Category | | | |
|---|---|---|---|Figure 2: Cytotoxicity comparison of control, bag A and bag B in incubator after 3 d bag test phase of Donor 1 - 3
1

## Slide 2
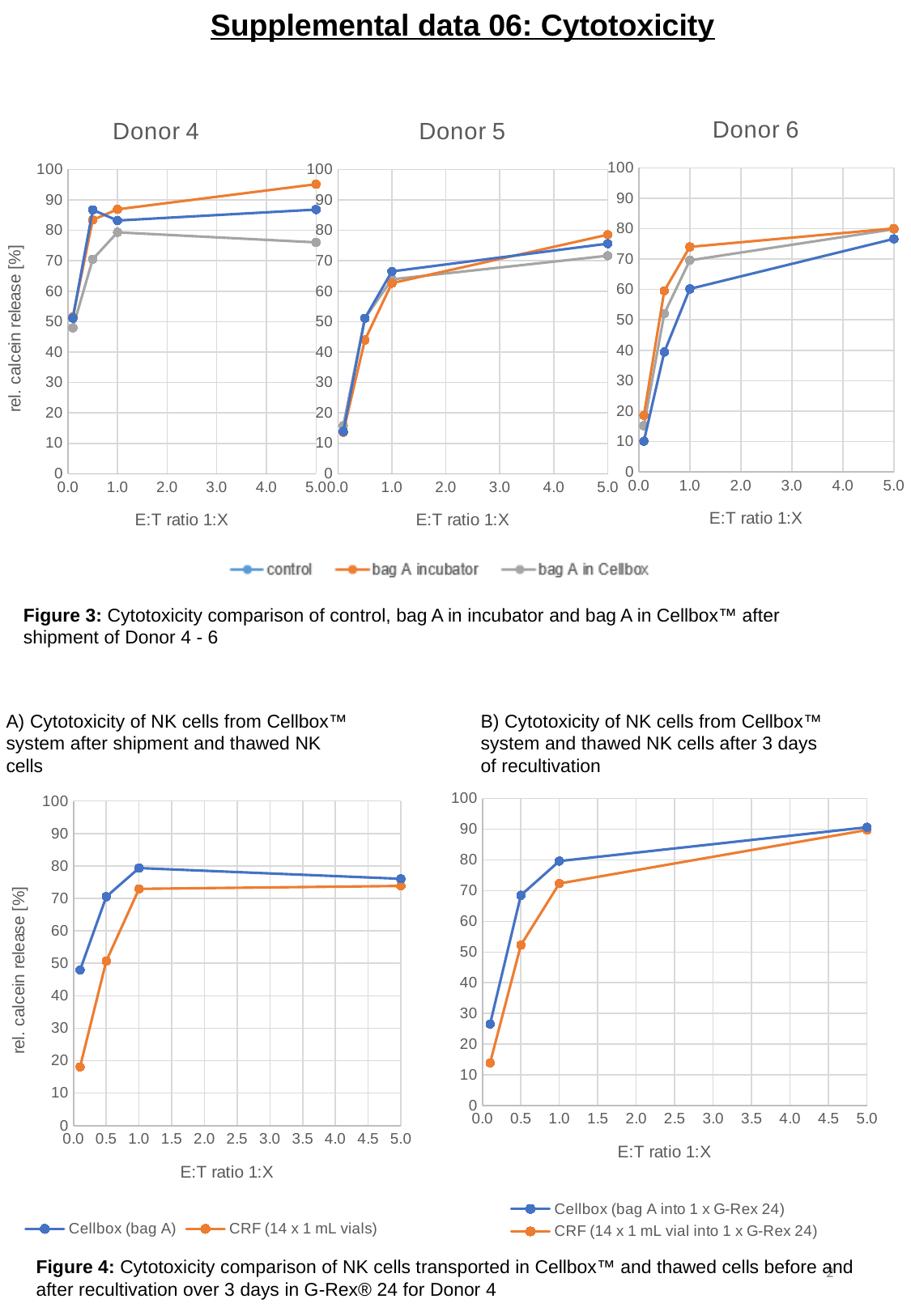

Supplemental data 06: Cytotoxicity
### Chart: Donor 6
| Category | | | |
|---|---|---|---|
### Chart: Donor 4
| Category | | | |
|---|---|---|---|
### Chart: Donor 5
| Category | | | |
|---|---|---|---|
Figure 3: Cytotoxicity comparison of control, bag A in incubator and bag A in Cellbox™ after shipment of Donor 4 - 6
A) Cytotoxicity of NK cells from Cellbox™ system after shipment and thawed NK cells
B) Cytotoxicity of NK cells from Cellbox™ system and thawed NK cells after 3 days of recultivation
### Chart
| Category | | |
|---|---|---|
### Chart
| Category | | |
|---|---|---|2
Figure 4: Cytotoxicity comparison of NK cells transported in Cellbox™ and thawed cells before and after recultivation over 3 days in G-Rex® 24 for Donor 4
