# Supplementary material for: “Live” cell shipment—a forward-looking transport option for cryo-sensitive cell-based therapies
Source: Front Bioeng Biotechnol. 2025 Dec 9;13:1706927. doi: 10.3389/fbioe.2025.1706927 (PMC12723144; doi:10.3389/fbioe.2025.1706927)
Supplement: Supplementary file 4 [file Presentation3.pptx]

## Slide 1
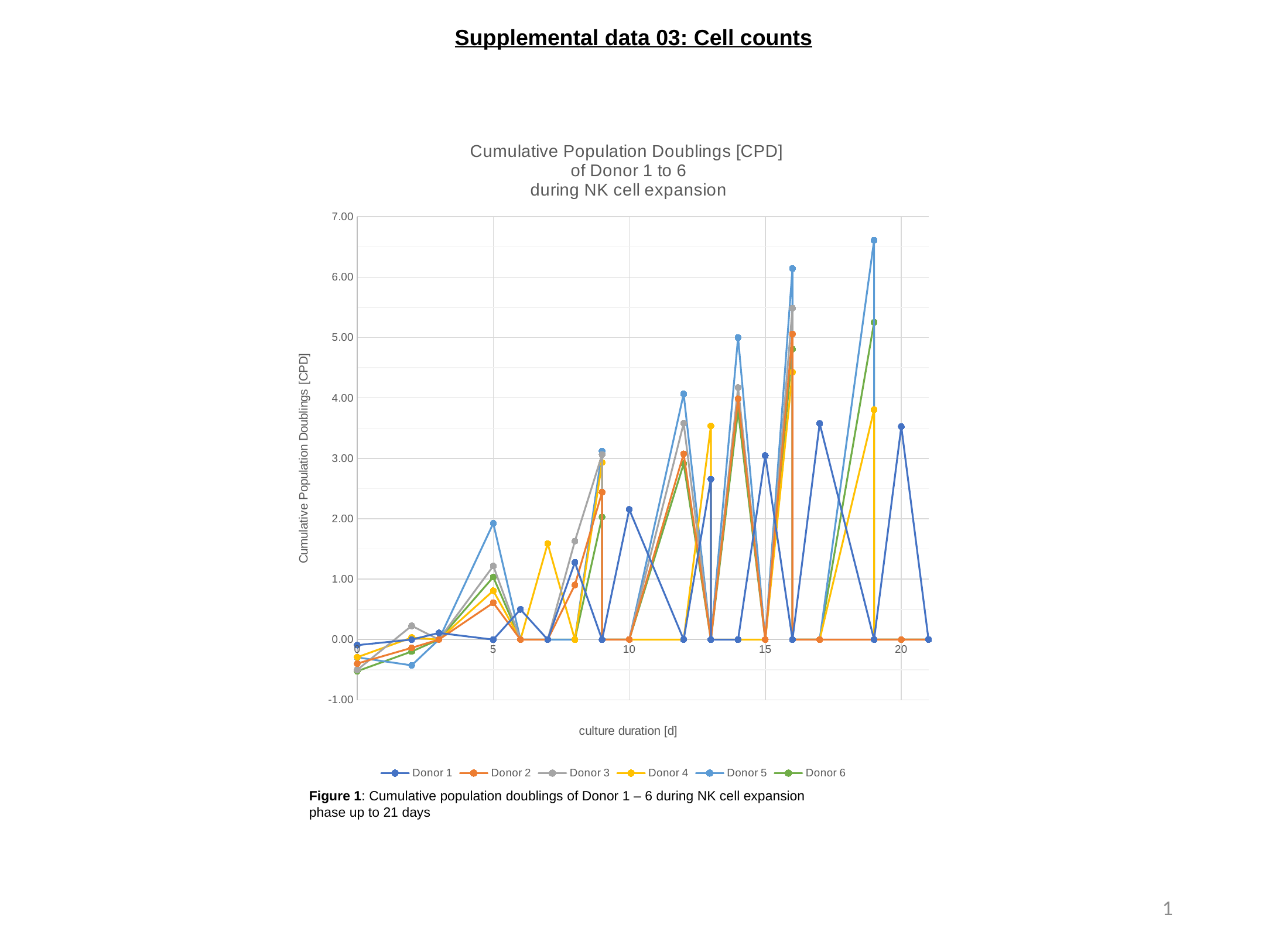

Supplemental data 03: Cell counts
### Chart: Cumulative Population Doublings [CPD]
of Donor 1 to 6
during NK cell expansion
| Category | | | | | | |
|---|---|---|---|---|---|---|Figure 1: Cumulative population doublings of Donor 1 – 6 during NK cell expansion phase up to 21 days
1

## Slide 2
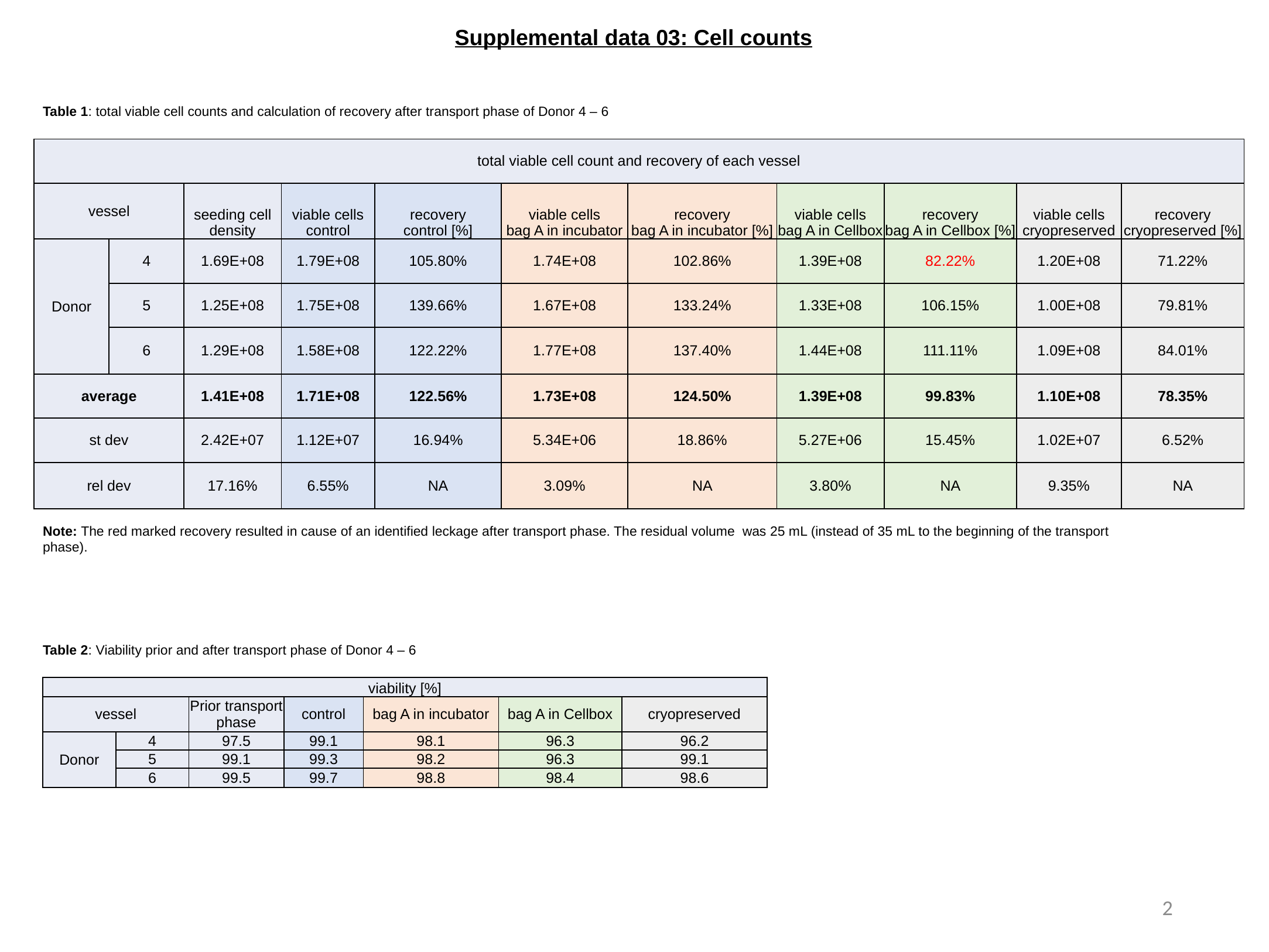

Supplemental data 03: Cell counts
Table 1: total viable cell counts and calculation of recovery after transport phase of Donor 4 – 6
| total viable cell count and recovery of each vessel | | | | | | | | | | |
| --- | --- | --- | --- | --- | --- | --- | --- | --- | --- | --- |
| vessel | | seeding cell density | viable cells control | recovery control [%] | viable cells bag A in incubator | recovery bag A in incubator [%] | viable cells bag A in Cellbox | recovery bag A in Cellbox [%] | viable cells cryopreserved | recovery cryopreserved [%] |
| Donor | 4 | 1.69E+08 | 1.79E+08 | 105.80% | 1.74E+08 | 102.86% | 1.39E+08 | 82.22% | 1.20E+08 | 71.22% |
| | 5 | 1.25E+08 | 1.75E+08 | 139.66% | 1.67E+08 | 133.24% | 1.33E+08 | 106.15% | 1.00E+08 | 79.81% |
| | 6 | 1.29E+08 | 1.58E+08 | 122.22% | 1.77E+08 | 137.40% | 1.44E+08 | 111.11% | 1.09E+08 | 84.01% |
| average | | 1.41E+08 | 1.71E+08 | 122.56% | 1.73E+08 | 124.50% | 1.39E+08 | 99.83% | 1.10E+08 | 78.35% |
| st dev | | 2.42E+07 | 1.12E+07 | 16.94% | 5.34E+06 | 18.86% | 5.27E+06 | 15.45% | 1.02E+07 | 6.52% |
| rel dev | | 17.16% | 6.55% | NA | 3.09% | NA | 3.80% | NA | 9.35% | NA |
Note: The red marked recovery resulted in cause of an identified leckage after transport phase. The residual volume was 25 mL (instead of 35 mL to the beginning of the transport phase).
Table 2: Viability prior and after transport phase of Donor 4 – 6
| viability [%] | | | | | | |
| --- | --- | --- | --- | --- | --- | --- |
| vessel | | Prior transport phase | control | bag A in incubator | bag A in Cellbox | cryopreserved |
| Donor | 4 | 97.5 | 99.1 | 98.1 | 96.3 | 96.2 |
| | 5 | 99.1 | 99.3 | 98.2 | 96.3 | 99.1 |
| | 6 | 99.5 | 99.7 | 98.8 | 98.4 | 98.6 |
2

## Slide 3
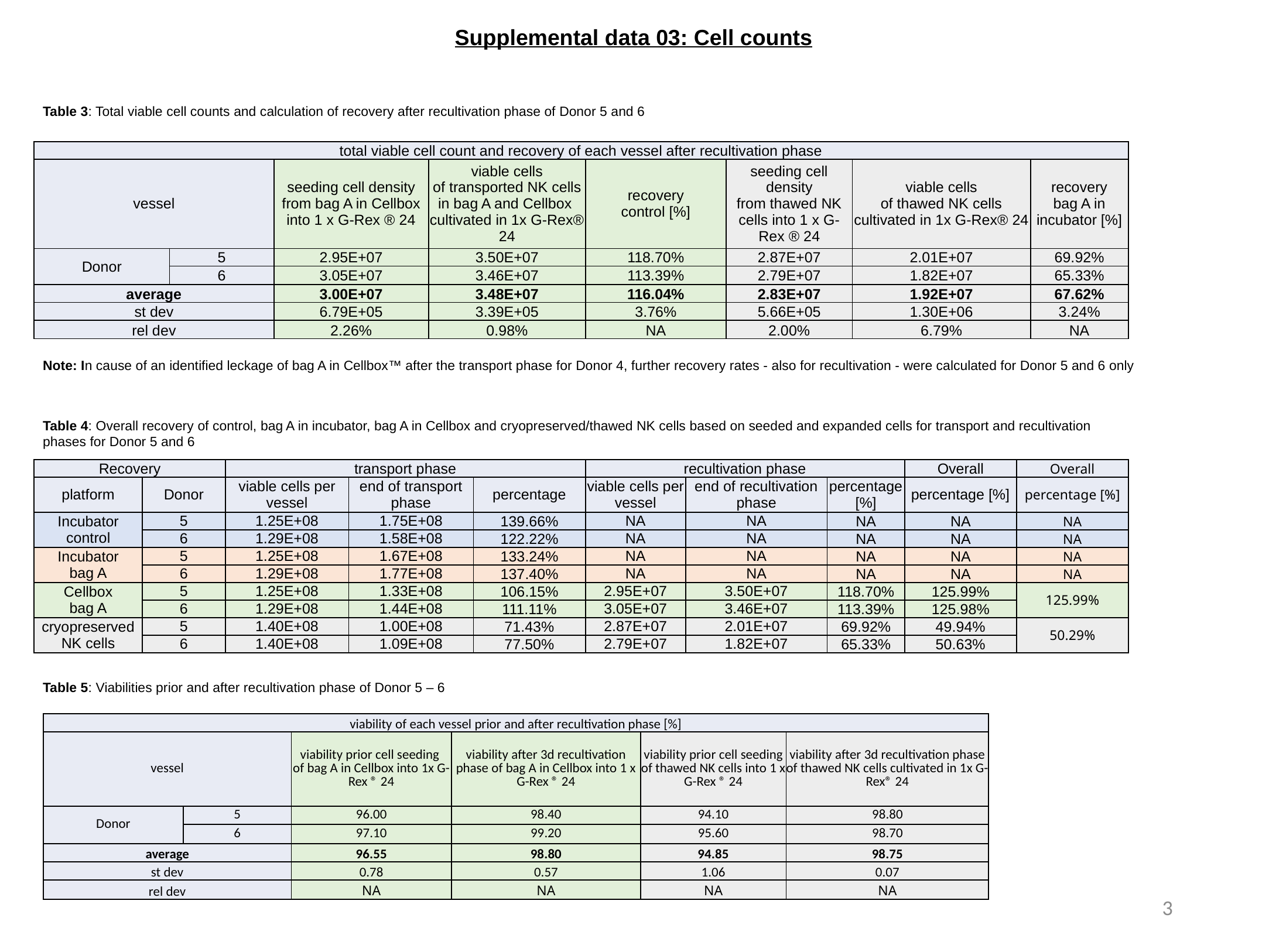

Supplemental data 03: Cell counts
Table 3: Total viable cell counts and calculation of recovery after recultivation phase of Donor 5 and 6
| total viable cell count and recovery of each vessel after recultivation phase | | | | | | | |
| --- | --- | --- | --- | --- | --- | --- | --- |
| vessel | | seeding cell density from bag A in Cellbox into 1 x G-Rex ® 24 | viable cells of transported NK cells in bag A and Cellbox cultivated in 1x G-Rex® 24 | recovery control [%] | seeding cell density from thawed NK cells into 1 x G-Rex ® 24 | viable cells of thawed NK cells cultivated in 1x G-Rex® 24 | recovery bag A in incubator [%] |
| Donor | 5 | 2.95E+07 | 3.50E+07 | 118.70% | 2.87E+07 | 2.01E+07 | 69.92% |
| | 6 | 3.05E+07 | 3.46E+07 | 113.39% | 2.79E+07 | 1.82E+07 | 65.33% |
| average | | 3.00E+07 | 3.48E+07 | 116.04% | 2.83E+07 | 1.92E+07 | 67.62% |
| st dev | | 6.79E+05 | 3.39E+05 | 3.76% | 5.66E+05 | 1.30E+06 | 3.24% |
| rel dev | | 2.26% | 0.98% | NA | 2.00% | 6.79% | NA |
Note: In cause of an identified leckage of bag A in Cellbox™ after the transport phase for Donor 4, further recovery rates - also for recultivation - were calculated for Donor 5 and 6 only
Table 4: Overall recovery of control, bag A in incubator, bag A in Cellbox and cryopreserved/thawed NK cells based on seeded and expanded cells for transport and recultivation phases for Donor 5 and 6
| Recovery | | transport phase | | | recultivation phase | | | Overall | Overall |
| --- | --- | --- | --- | --- | --- | --- | --- | --- | --- |
| platform | Donor | viable cells per vessel | end of transport phase | percentage | viable cells per vessel | end of recultivation phase | percentage [%] | percentage [%] | percentage [%] |
| Incubator control | 5 | 1.25E+08 | 1.75E+08 | 139.66% | NA | NA | NA | NA | NA |
| | 6 | 1.29E+08 | 1.58E+08 | 122.22% | NA | NA | NA | NA | NA |
| Incubator bag A | 5 | 1.25E+08 | 1.67E+08 | 133.24% | NA | NA | NA | NA | NA |
| | 6 | 1.29E+08 | 1.77E+08 | 137.40% | NA | NA | NA | NA | NA |
| Cellbox bag A | 5 | 1.25E+08 | 1.33E+08 | 106.15% | 2.95E+07 | 3.50E+07 | 118.70% | 125.99% | 125.99% |
| | 6 | 1.29E+08 | 1.44E+08 | 111.11% | 3.05E+07 | 3.46E+07 | 113.39% | 125.98% | |
| cryopreserved NK cells | 5 | 1.40E+08 | 1.00E+08 | 71.43% | 2.87E+07 | 2.01E+07 | 69.92% | 49.94% | 50.29% |
| | 6 | 1.40E+08 | 1.09E+08 | 77.50% | 2.79E+07 | 1.82E+07 | 65.33% | 50.63% | |
Table 5: Viabilities prior and after recultivation phase of Donor 5 – 6
| viability of each vessel prior and after recultivation phase [%] | | | | | |
| --- | --- | --- | --- | --- | --- |
| vessel | | viability prior cell seeding of bag A in Cellbox into 1x G-Rex ® 24 | viability after 3d recultivation phase of bag A in Cellbox into 1 x G-Rex ® 24 | viability prior cell seeding of thawed NK cells into 1 x G-Rex ® 24 | viability after 3d recultivation phase of thawed NK cells cultivated in 1x G-Rex® 24 |
| Donor | 5 | 96.00 | 98.40 | 94.10 | 98.80 |
| | 6 | 97.10 | 99.20 | 95.60 | 98.70 |
| average | | 96.55 | 98.80 | 94.85 | 98.75 |
| st dev | | 0.78 | 0.57 | 1.06 | 0.07 |
| rel dev | | NA | NA | NA | NA |
3
