# Supplementary material for: “Live” cell shipment—a forward-looking transport option for cryo-sensitive cell-based therapies
Source: Front Bioeng Biotechnol. 2025 Dec 9;13:1706927. doi: 10.3389/fbioe.2025.1706927 (PMC12723144; doi:10.3389/fbioe.2025.1706927)
Supplement: Supplementary file 5 [file Presentation2.pptx]

## Slide 1
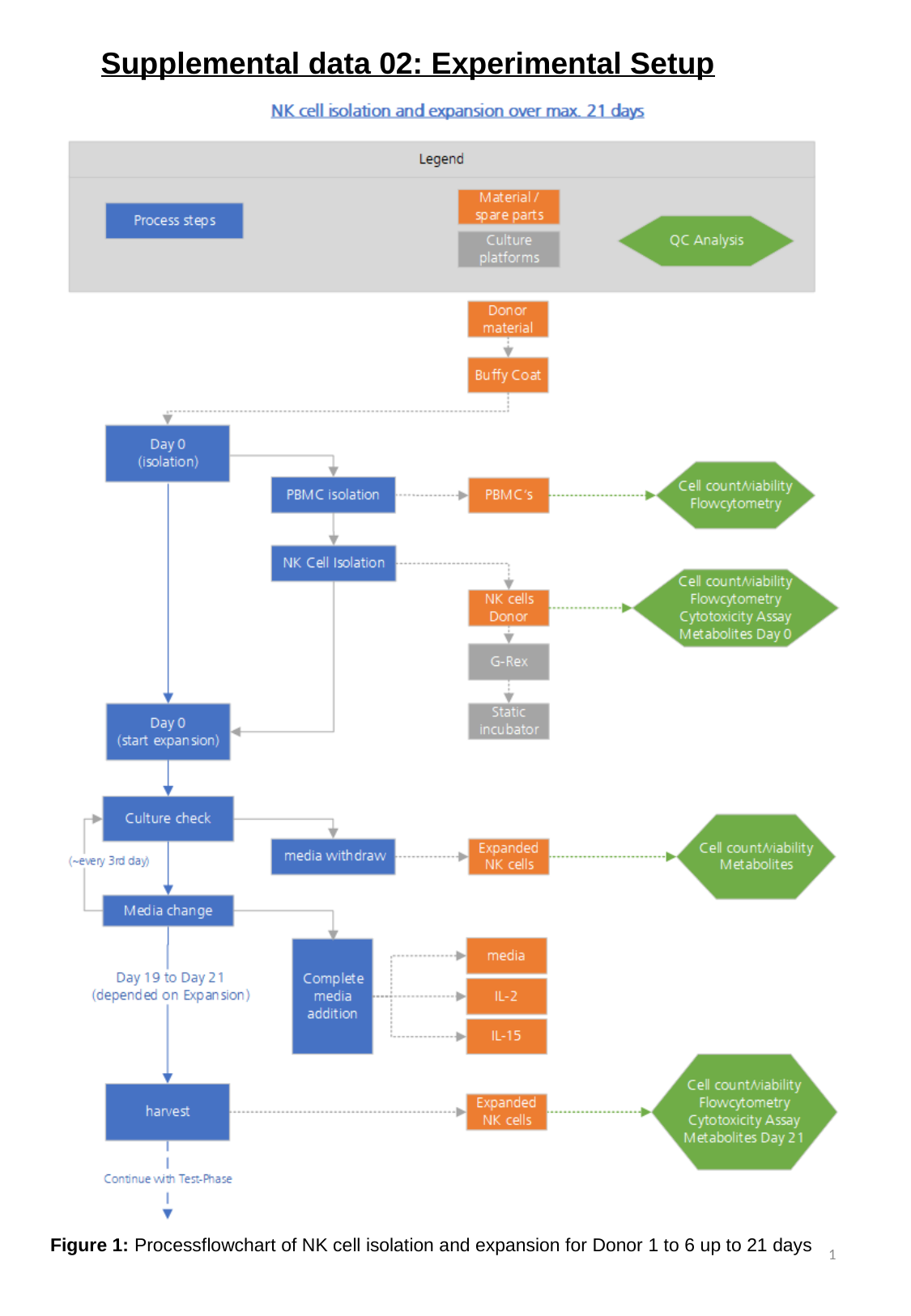

Supplemental data 02: Experimental Setup
1
Figure 1: Processflowchart of NK cell isolation and expansion for Donor 1 to 6 up to 21 days

## Slide 2
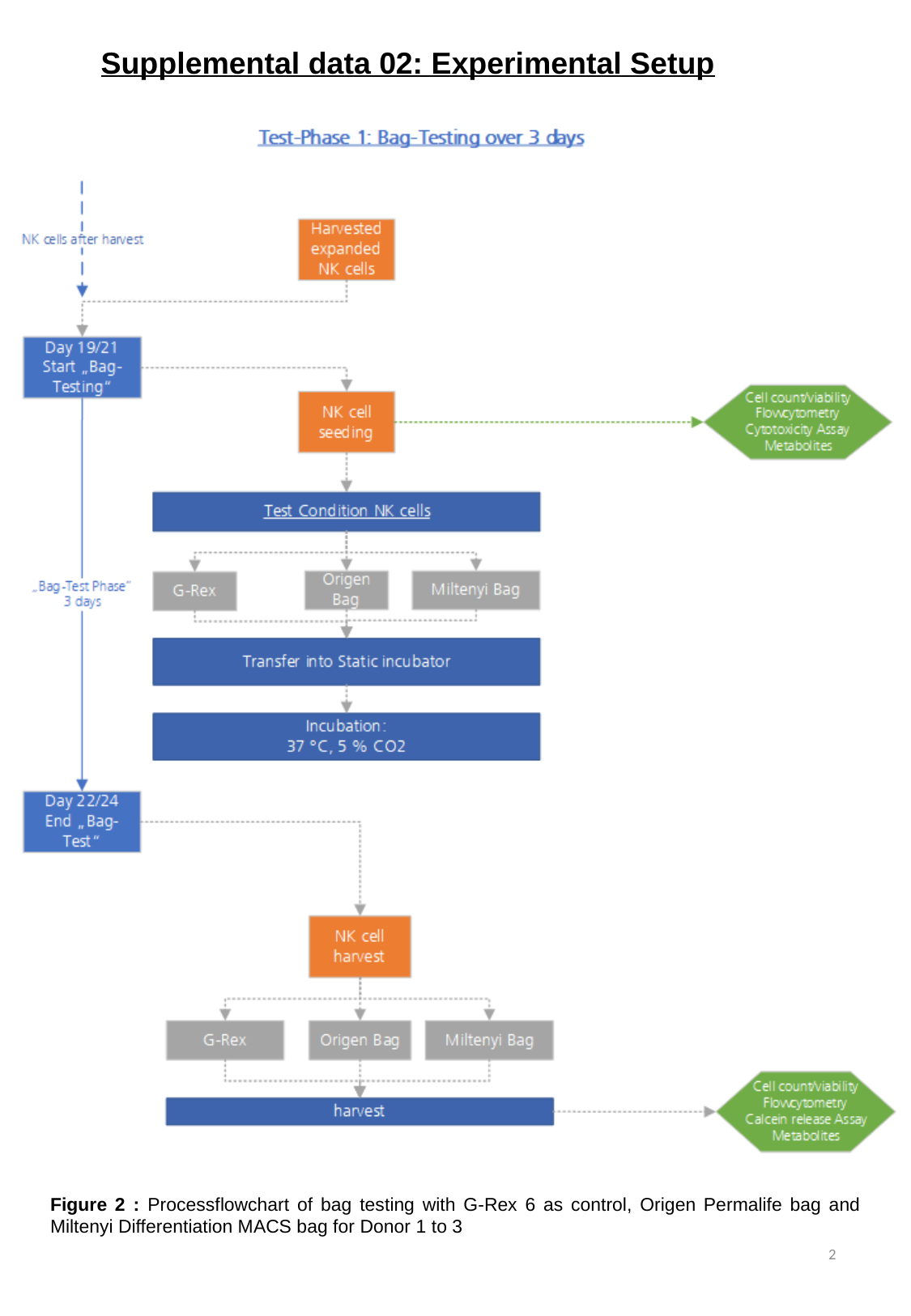

Supplemental data 02: Experimental Setup
Figure 2 : Processflowchart of bag testing with G-Rex 6 as control, Origen Permalife bag and Miltenyi Differentiation MACS bag for Donor 1 to 3
2

## Slide 3
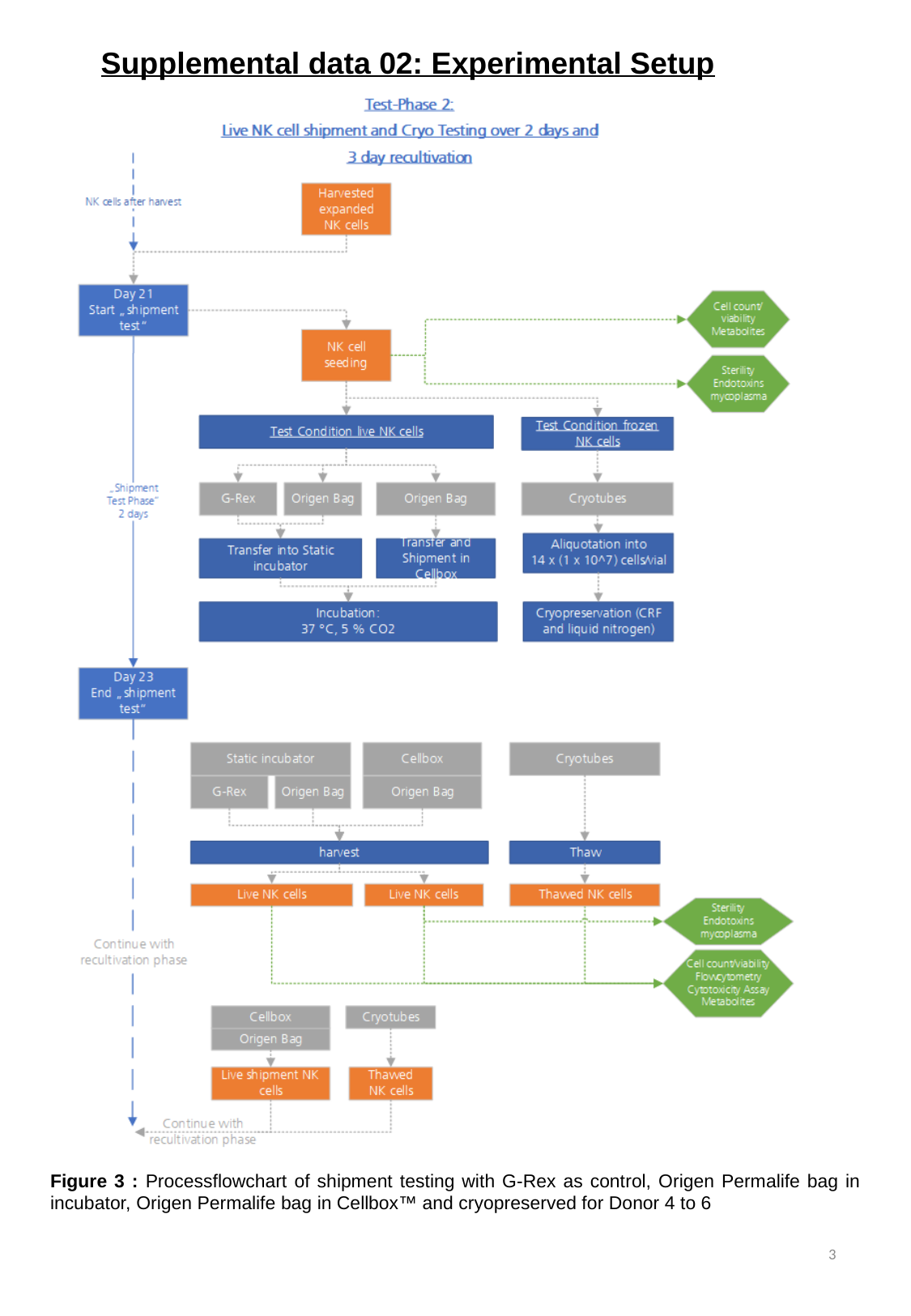

Supplemental data 02: Experimental Setup
Figure 3 : Processflowchart of shipment testing with G-Rex as control, Origen Permalife bag in incubator, Origen Permalife bag in Cellbox™ and cryopreserved for Donor 4 to 6
3
