# Supplementary material for: “Live” cell shipment—a forward-looking transport option for cryo-sensitive cell-based therapies
Source: Front Bioeng Biotechnol. 2025 Dec 9;13:1706927. doi: 10.3389/fbioe.2025.1706927 (PMC12723144; doi:10.3389/fbioe.2025.1706927)
Supplement: Supplementary file 6 [file Presentation5.pptx]

## Slide 1
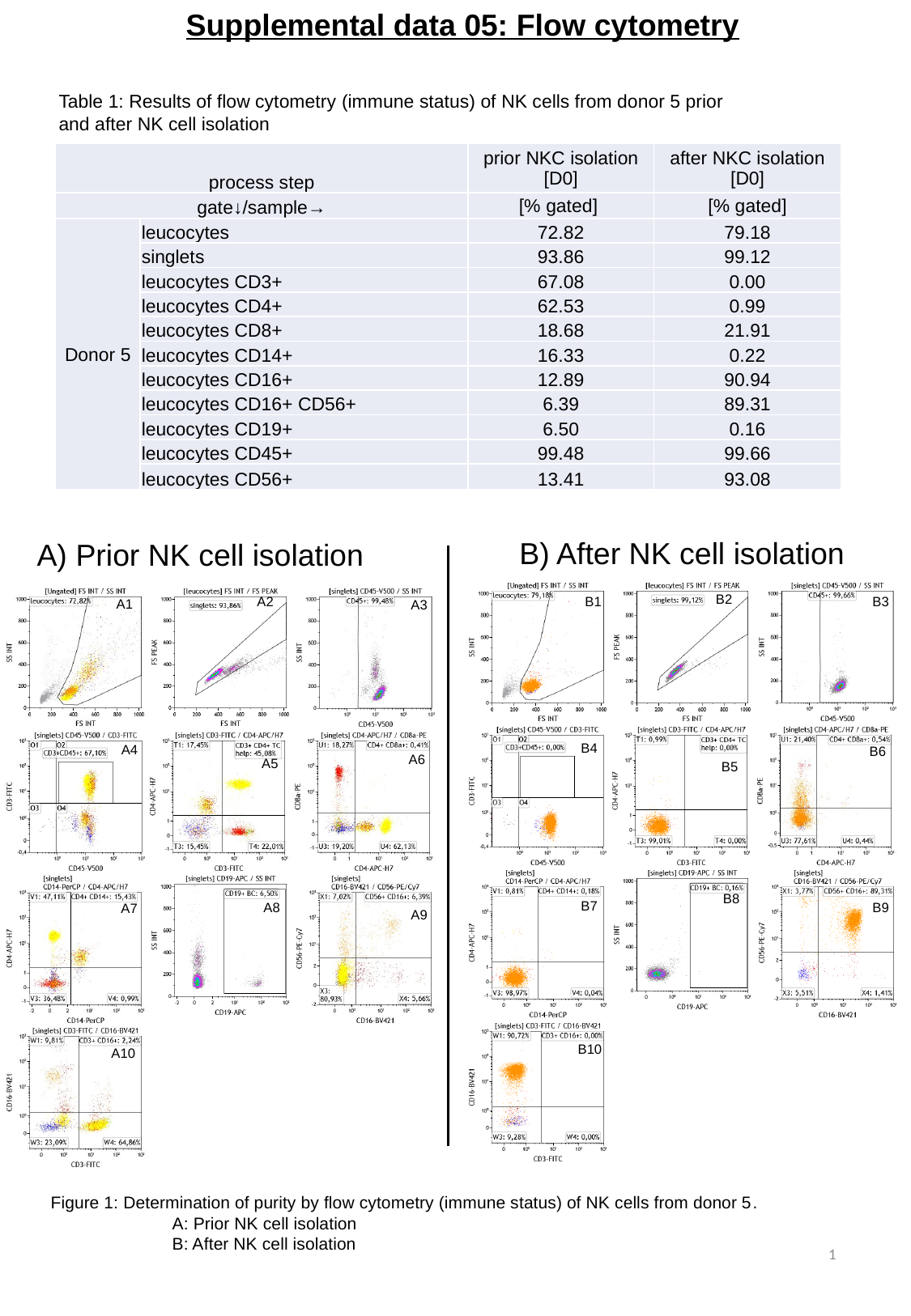

Supplemental data 05: Flow cytometry
Table 1: Results of flow cytometry (immune status) of NK cells from donor 5 prior
and after NK cell isolation
| process step | | prior NKC isolation [D0] | after NKC isolation [D0] |
| --- | --- | --- | --- |
| gate↓/sample→ | | [% gated] | [% gated] |
| Donor 5 | leucocytes | 72.82 | 79.18 |
| | singlets | 93.86 | 99.12 |
| | leucocytes CD3+ | 67.08 | 0.00 |
| | leucocytes CD4+ | 62.53 | 0.99 |
| | leucocytes CD8+ | 18.68 | 21.91 |
| | leucocytes CD14+ | 16.33 | 0.22 |
| | leucocytes CD16+ | 12.89 | 90.94 |
| | leucocytes CD16+ CD56+ | 6.39 | 89.31 |
| | leucocytes CD19+ | 6.50 | 0.16 |
| | leucocytes CD45+ | 99.48 | 99.66 |
| | leucocytes CD56+ | 13.41 | 93.08 |
B) After NK cell isolation
A) Prior NK cell isolation
B2
A2
B1
B3
A1
A3
B4
A4
B6
A6
A5
B5
B8
B7
A8
B9
A7
A9
B10
A10
Figure 1: Determination of purity by flow cytometry (immune status) of NK cells from donor 5.
	A: Prior NK cell isolation
	B: After NK cell isolation
1

## Slide 2
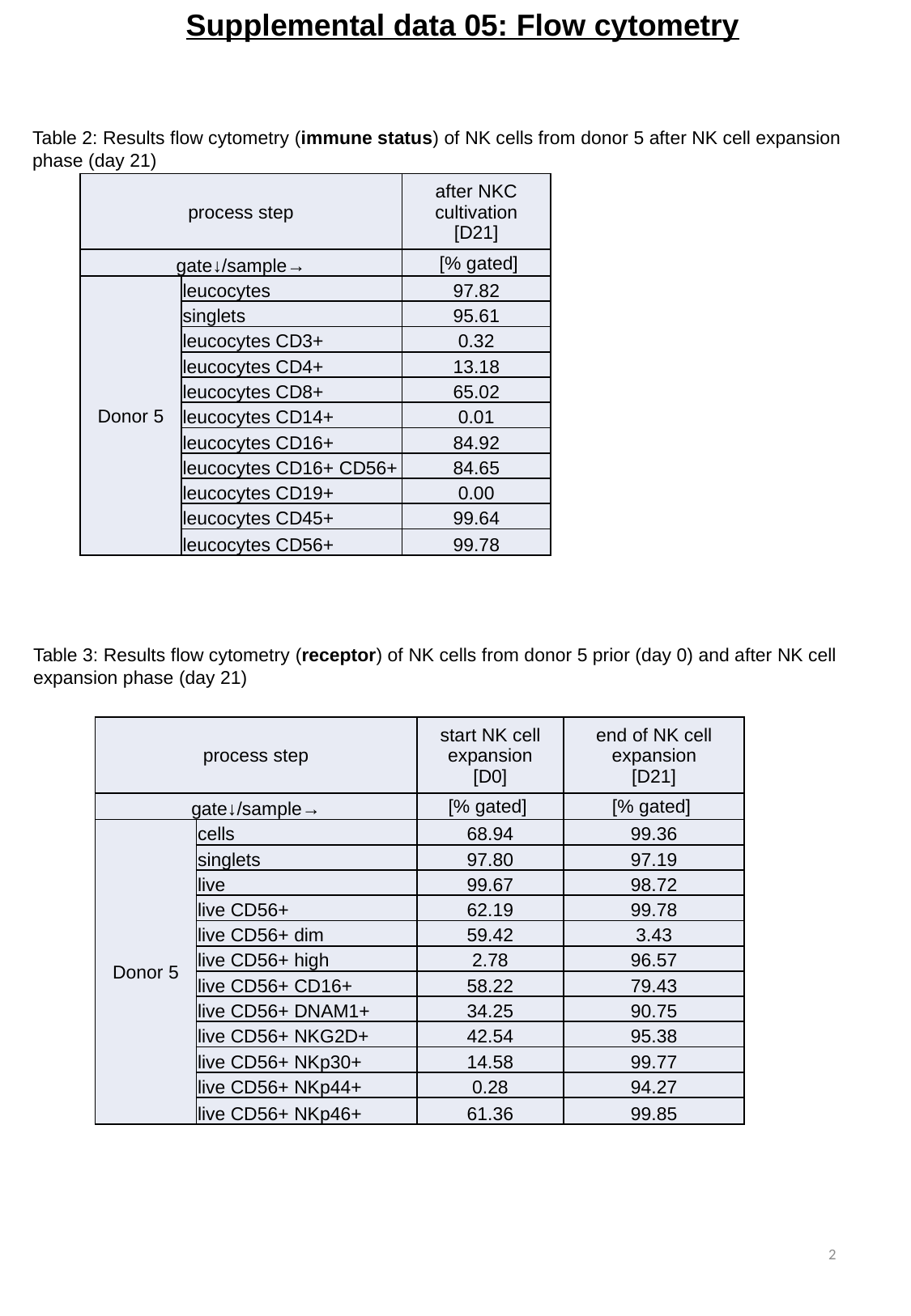

Supplemental data 05: Flow cytometry
Table 2: Results flow cytometry (immune status) of NK cells from donor 5 after NK cell expansion
phase (day 21)
| process step | | after NKC cultivation [D21] |
| --- | --- | --- |
| gate↓/sample→ | | [% gated] |
| Donor 5 | leucocytes | 97.82 |
| | singlets | 95.61 |
| | leucocytes CD3+ | 0.32 |
| | leucocytes CD4+ | 13.18 |
| | leucocytes CD8+ | 65.02 |
| | leucocytes CD14+ | 0.01 |
| | leucocytes CD16+ | 84.92 |
| | leucocytes CD16+ CD56+ | 84.65 |
| | leucocytes CD19+ | 0.00 |
| | leucocytes CD45+ | 99.64 |
| | leucocytes CD56+ | 99.78 |
Table 3: Results flow cytometry (receptor) of NK cells from donor 5 prior (day 0) and after NK cell expansion phase (day 21)
| process step | | start NK cell expansion [D0] | end of NK cell expansion [D21] |
| --- | --- | --- | --- |
| gate↓/sample→ | | [% gated] | [% gated] |
| Donor 5 | cells | 68.94 | 99.36 |
| | singlets | 97.80 | 97.19 |
| | live | 99.67 | 98.72 |
| | live CD56+ | 62.19 | 99.78 |
| | live CD56+ dim | 59.42 | 3.43 |
| | live CD56+ high | 2.78 | 96.57 |
| | live CD56+ CD16+ | 58.22 | 79.43 |
| | live CD56+ DNAM1+ | 34.25 | 90.75 |
| | live CD56+ NKG2D+ | 42.54 | 95.38 |
| | live CD56+ NKp30+ | 14.58 | 99.77 |
| | live CD56+ NKp44+ | 0.28 | 94.27 |
| | live CD56+ NKp46+ | 61.36 | 99.85 |
2

## Slide 3
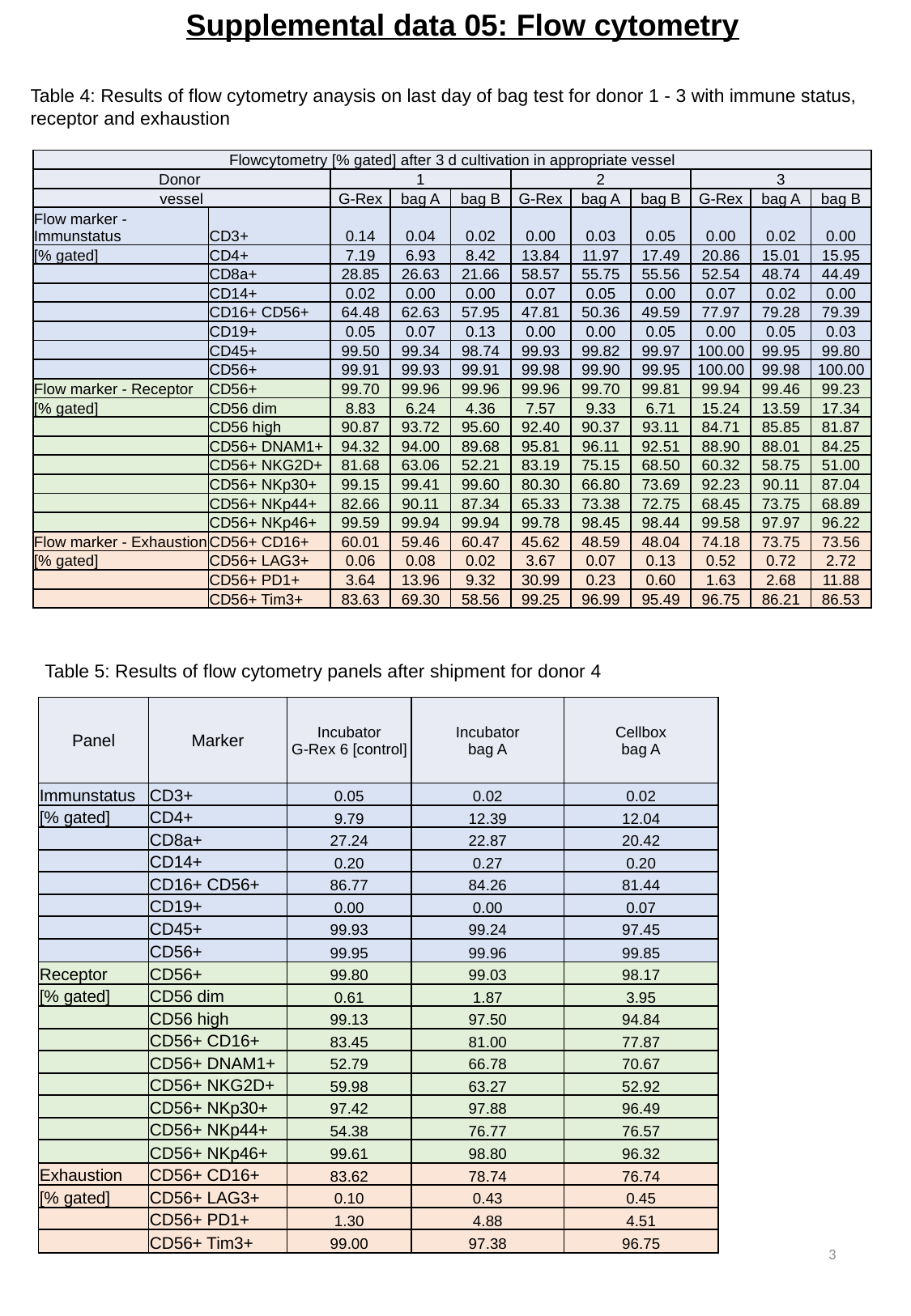

Supplemental data 05: Flow cytometry
Table 4: Results of flow cytometry anaysis on last day of bag test for donor 1 - 3 with immune status, receptor and exhaustion
| Flowcytometry [% gated] after 3 d cultivation in appropriate vessel | | | | | | | | | | |
| --- | --- | --- | --- | --- | --- | --- | --- | --- | --- | --- |
| Donor | | 1 | | | 2 | | | 3 | | |
| vessel | | G-Rex | bag A | bag B | G-Rex | bag A | bag B | G-Rex | bag A | bag B |
| Flow marker - Immunstatus | CD3+ | 0.14 | 0.04 | 0.02 | 0.00 | 0.03 | 0.05 | 0.00 | 0.02 | 0.00 |
| [% gated] | CD4+ | 7.19 | 6.93 | 8.42 | 13.84 | 11.97 | 17.49 | 20.86 | 15.01 | 15.95 |
| | CD8a+ | 28.85 | 26.63 | 21.66 | 58.57 | 55.75 | 55.56 | 52.54 | 48.74 | 44.49 |
| | CD14+ | 0.02 | 0.00 | 0.00 | 0.07 | 0.05 | 0.00 | 0.07 | 0.02 | 0.00 |
| | CD16+ CD56+ | 64.48 | 62.63 | 57.95 | 47.81 | 50.36 | 49.59 | 77.97 | 79.28 | 79.39 |
| | CD19+ | 0.05 | 0.07 | 0.13 | 0.00 | 0.00 | 0.05 | 0.00 | 0.05 | 0.03 |
| | CD45+ | 99.50 | 99.34 | 98.74 | 99.93 | 99.82 | 99.97 | 100.00 | 99.95 | 99.80 |
| | CD56+ | 99.91 | 99.93 | 99.91 | 99.98 | 99.90 | 99.95 | 100.00 | 99.98 | 100.00 |
| Flow marker - Receptor | CD56+ | 99.70 | 99.96 | 99.96 | 99.96 | 99.70 | 99.81 | 99.94 | 99.46 | 99.23 |
| [% gated] | CD56 dim | 8.83 | 6.24 | 4.36 | 7.57 | 9.33 | 6.71 | 15.24 | 13.59 | 17.34 |
| | CD56 high | 90.87 | 93.72 | 95.60 | 92.40 | 90.37 | 93.11 | 84.71 | 85.85 | 81.87 |
| | CD56+ DNAM1+ | 94.32 | 94.00 | 89.68 | 95.81 | 96.11 | 92.51 | 88.90 | 88.01 | 84.25 |
| | CD56+ NKG2D+ | 81.68 | 63.06 | 52.21 | 83.19 | 75.15 | 68.50 | 60.32 | 58.75 | 51.00 |
| | CD56+ NKp30+ | 99.15 | 99.41 | 99.60 | 80.30 | 66.80 | 73.69 | 92.23 | 90.11 | 87.04 |
| | CD56+ NKp44+ | 82.66 | 90.11 | 87.34 | 65.33 | 73.38 | 72.75 | 68.45 | 73.75 | 68.89 |
| | CD56+ NKp46+ | 99.59 | 99.94 | 99.94 | 99.78 | 98.45 | 98.44 | 99.58 | 97.97 | 96.22 |
| Flow marker - Exhaustion | CD56+ CD16+ | 60.01 | 59.46 | 60.47 | 45.62 | 48.59 | 48.04 | 74.18 | 73.75 | 73.56 |
| [% gated] | CD56+ LAG3+ | 0.06 | 0.08 | 0.02 | 3.67 | 0.07 | 0.13 | 0.52 | 0.72 | 2.72 |
| | CD56+ PD1+ | 3.64 | 13.96 | 9.32 | 30.99 | 0.23 | 0.60 | 1.63 | 2.68 | 11.88 |
| | CD56+ Tim3+ | 83.63 | 69.30 | 58.56 | 99.25 | 96.99 | 95.49 | 96.75 | 86.21 | 86.53 |
Table 5: Results of flow cytometry panels after shipment for donor 4
| Panel | Marker | Incubator G-Rex 6 [control] | Incubator bag A | Cellbox bag A |
| --- | --- | --- | --- | --- |
| Immunstatus | CD3+ | 0.05 | 0.02 | 0.02 |
| [% gated] | CD4+ | 9.79 | 12.39 | 12.04 |
| | CD8a+ | 27.24 | 22.87 | 20.42 |
| | CD14+ | 0.20 | 0.27 | 0.20 |
| | CD16+ CD56+ | 86.77 | 84.26 | 81.44 |
| | CD19+ | 0.00 | 0.00 | 0.07 |
| | CD45+ | 99.93 | 99.24 | 97.45 |
| | CD56+ | 99.95 | 99.96 | 99.85 |
| Receptor | CD56+ | 99.80 | 99.03 | 98.17 |
| [% gated] | CD56 dim | 0.61 | 1.87 | 3.95 |
| | CD56 high | 99.13 | 97.50 | 94.84 |
| | CD56+ CD16+ | 83.45 | 81.00 | 77.87 |
| | CD56+ DNAM1+ | 52.79 | 66.78 | 70.67 |
| | CD56+ NKG2D+ | 59.98 | 63.27 | 52.92 |
| | CD56+ NKp30+ | 97.42 | 97.88 | 96.49 |
| | CD56+ NKp44+ | 54.38 | 76.77 | 76.57 |
| | CD56+ NKp46+ | 99.61 | 98.80 | 96.32 |
| Exhaustion | CD56+ CD16+ | 83.62 | 78.74 | 76.74 |
| [% gated] | CD56+ LAG3+ | 0.10 | 0.43 | 0.45 |
| | CD56+ PD1+ | 1.30 | 4.88 | 4.51 |
| | CD56+ Tim3+ | 99.00 | 97.38 | 96.75 |
3

## Slide 4
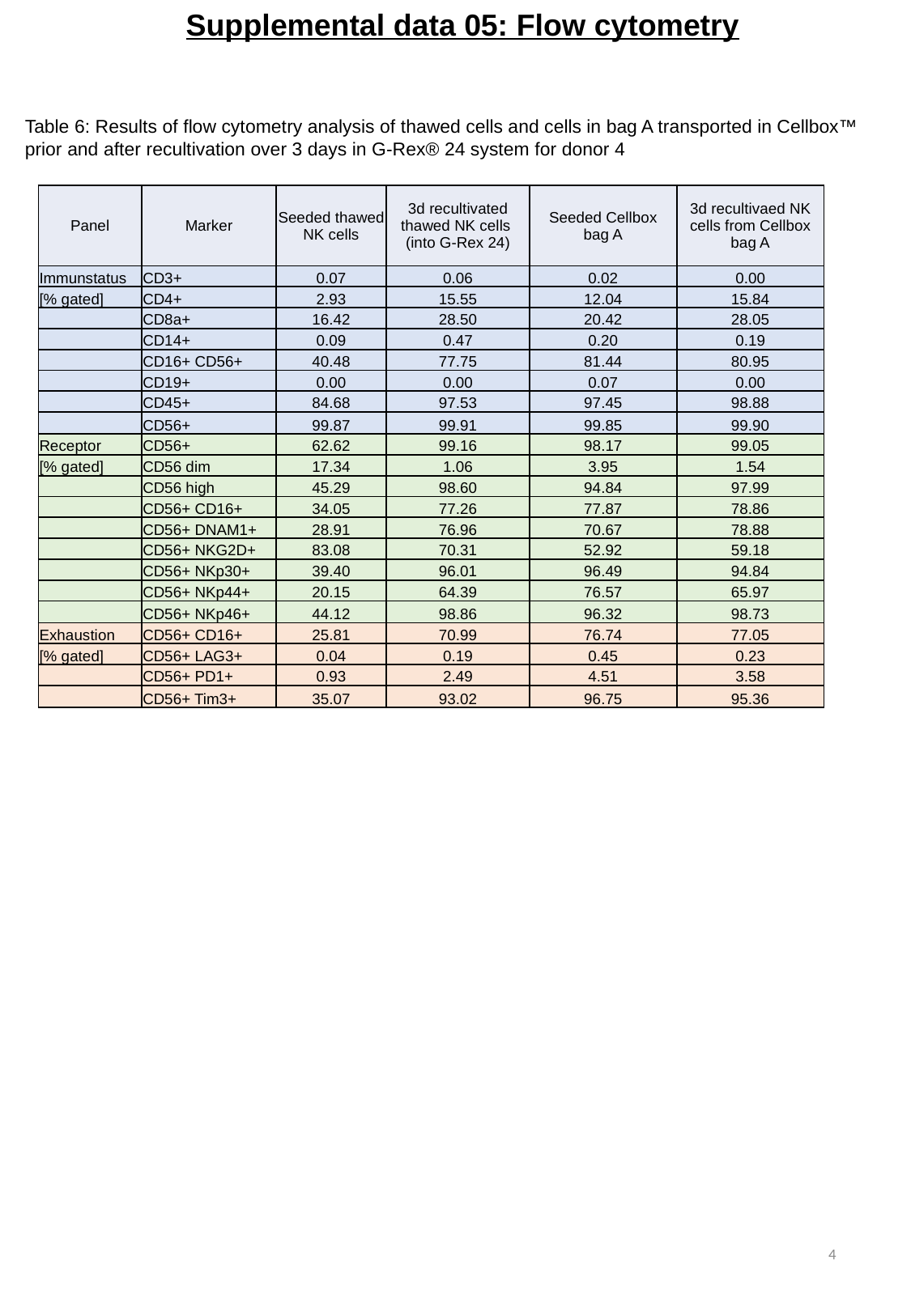

Supplemental data 05: Flow cytometry
Table 6: Results of flow cytometry analysis of thawed cells and cells in bag A transported in Cellbox™ prior and after recultivation over 3 days in G-Rex® 24 system for donor 4
| Panel | Marker | Seeded thawed NK cells | 3d recultivated thawed NK cells (into G-Rex 24) | Seeded Cellbox bag A | 3d recultivaed NK cells from Cellbox bag A |
| --- | --- | --- | --- | --- | --- |
| Immunstatus | CD3+ | 0.07 | 0.06 | 0.02 | 0.00 |
| [% gated] | CD4+ | 2.93 | 15.55 | 12.04 | 15.84 |
| | CD8a+ | 16.42 | 28.50 | 20.42 | 28.05 |
| | CD14+ | 0.09 | 0.47 | 0.20 | 0.19 |
| | CD16+ CD56+ | 40.48 | 77.75 | 81.44 | 80.95 |
| | CD19+ | 0.00 | 0.00 | 0.07 | 0.00 |
| | CD45+ | 84.68 | 97.53 | 97.45 | 98.88 |
| | CD56+ | 99.87 | 99.91 | 99.85 | 99.90 |
| Receptor | CD56+ | 62.62 | 99.16 | 98.17 | 99.05 |
| [% gated] | CD56 dim | 17.34 | 1.06 | 3.95 | 1.54 |
| | CD56 high | 45.29 | 98.60 | 94.84 | 97.99 |
| | CD56+ CD16+ | 34.05 | 77.26 | 77.87 | 78.86 |
| | CD56+ DNAM1+ | 28.91 | 76.96 | 70.67 | 78.88 |
| | CD56+ NKG2D+ | 83.08 | 70.31 | 52.92 | 59.18 |
| | CD56+ NKp30+ | 39.40 | 96.01 | 96.49 | 94.84 |
| | CD56+ NKp44+ | 20.15 | 64.39 | 76.57 | 65.97 |
| | CD56+ NKp46+ | 44.12 | 98.86 | 96.32 | 98.73 |
| Exhaustion | CD56+ CD16+ | 25.81 | 70.99 | 76.74 | 77.05 |
| [% gated] | CD56+ LAG3+ | 0.04 | 0.19 | 0.45 | 0.23 |
| | CD56+ PD1+ | 0.93 | 2.49 | 4.51 | 3.58 |
| | CD56+ Tim3+ | 35.07 | 93.02 | 96.75 | 95.36 |
4
